# The Use of Prescription Drugs, Recreational Drugs, and “Soft Enhancers” for Cognitive Enhancement among Swiss Secondary School Students

**DOI:** 10.1371/journal.pone.0141289

**Published:** 2015-10-27

**Authors:** Evangelia Liakoni, Michael P. Schaub, Larissa J. Maier, Gaëlle-Vanessa Glauser, Matthias E. Liechti

**Affiliations:** 1 Department of Clinical Research, Division of Clinical Pharmacology and Toxicology, University Hospital, Basel, Switzerland; 2 Swiss Research Institute for Public Health and Addiction (ISGF), Associated Institute at the University of Zurich and WHO Collaborating Centre, Zurich, Switzerland; Penn State College of Medicine, UNITED STATES

## Abstract

The use of prescription or recreational drugs for cognitive enhancement (CE) is prevalent among students. However, the prevalence of CE among Swiss school students is unknown. We therefore performed a cross-sectional online survey including ≥ 16-year-old students from bridge-year schools (10^th^ grade), vocational schools, and upper secondary schools (10^th^-12^th^ grade) in the Canton of Zurich to investigate the prevalence of and motives for the use of prescription drugs, recreational drugs, and/or freely available soft enhancers for CE. A total of 1,139 students were included. Of these, 54.5% reported the use of prescription drugs (9.2%), recreational drugs including alcohol (6.2%), or soft enhancers (51.3%) explicitly for CE at least once in their lives. The last-year and last-month prevalence for CE considering all substances was 45.5% and 39.5%, respectively. Soft enhancers were the substances that were most commonly used (ever, last-year, and last-month, respectively), including energy drinks (33.3%, 28.4%, and 24.6%), coffee (29.8%, 25.1%, and 21.9%), and tobacco (12.6%, 9.3%, and 8.3%). CE with methylphenidate was less prevalent (4.0%, 2.8%, and 2.0%). However, the use of prescription drugs, alcohol, or illegal drugs for CE was reported by 13.3% of the participants. The most common motives for use were to stay awake and improve concentration. CE was more prevalent among students who reported higher levels of stress or performance pressure and students with psychiatric disorders. In conclusion, half of the school students had used a substance at least once in their lives to improve school performance. Soft enhancers were most commonly used. Prevalence rates were similar to those reported by Swiss university students, indicating that the use of prescription or recreational drugs for CE already occurs before starting higher education. Performance pressure, stress, and psychiatric disorders may be associated with CE.

## Introduction

Pharmacological cognitive enhancement (CE) refers to the nonmedical use of prescription drugs and recreational drugs to improve performance while studying [1–5]. The use of prescription drugs (e.g., methylphenidate and modafinil) is mainly considered in studies or discussions on CE [6,7]. However, other substances, such as recreational drugs (e.g., cocaine and amphetamine) [2] and “soft enhancers” [2,8] are also included. Soft enhancers are readily available substances, such as coffee, caffeine tablets, energy drinks, herbal drugs, vitamins, and tonics, that can be used for CE [2,8]. Typically, substances with stimulant properties are used for “direct” CE. However, “indirect” CE with sedative substances (e.g., hypnotics and alcohol) are also taken to prepare for or cope with stressful situations, in which they are used to improve sleep or relax before exams or improve studying the next day [2,9].

There are numerous concerns regarding the use of prescription or recreational drugs for CE. The benefits in terms of efficacy are likely small [4,7,10,11], and the harm-benefit ratio is unclear for medications that are marketed for patients but used by healthy people [5,11–13]. Moreover, students may perceive peer pressure (coercion) to use various substances for CE [12] or consider CE unfair and cheating [5,12,14–16], similar to doping in sports [17]. The first empirical data on prescription drug misuse for CE were provided in the United States [18–20]. More recently, several European studies examined the prevalence of CE among university students and school students [9,21,22]. In Switzerland, two online surveys recently investigated the prevalence rates of CE among Swiss university students [2,12], showing that a significant proportion of Swiss university students had used prescription drugs (6.2–7.6%) or recreational drugs including alcohol (7.8%) for CE. However, data from younger Swiss school students are lacking. Therefore, we performed a survey among 55 schools in the Canton of Zurich to provide estimates of substance use for CE among school students. We also explored whether performance pressure, stress, and psychiatric disorders are associated with substance use for CE.

## Methods

### Survey and participants

This cross-sectional study was conducted during a 3-month period between June 12, 2014, and September 12, 2014, in the Canton of Zurich, Switzerland. The study was approved by the ethics committee of the Philosophical Faculty of the University of Zurich and the educational authorities in the Canton of Zurich (Mittelschul- und Berufsbildungsamt des Kantons Zürich). The study was supported by the Office for Alcohol and Drug Misuse Prevention in the Canton of Zurich (Zürcher Fachstelle zur Prävention des Alkohol- und Medikamenten-Missbrauchs). The minimum age of the participants was set by the ethic committee at 16 years because the participation of younger students would have required additional parental consent. No parental consent was required for minor students older than 16 years for this non-interventional survey-type study in Switzerland. Participation was voluntary and anonymous. No incentive was provided for participation in the study. The participants had the right to withdraw from the study at any time without consequences. To ensure standardized administration and a good response rate and thus more valid data, the survey was conducted in computer labs at the schools in the presence of teachers.

The administrations of 55 schools with an estimated 32,000 potential participants (eleven 10^th^ grade bridge-year program schools [*Berufsvorbereitungsschulen*, *zehntes Schuljahr*], an additional year offered to adolescents who do not start vocational or upper secondary education immediately after completing lower secondary education, 25 vocational schools [*Berufsfachschulen*], and 19 upper secondary schools [10^th^-12^th^ grade; *Mittelschulen*, also named *Gymnasium* or *Kantonsschulen*]) were contacted by e-mail or letters to explain the rationale for the study and the study procedures. The contact information of 154 teachers was obtained, providing access to an estimated number of 2,310 potential participants. Thus, participants were included only if the teacher supported the administration of the questionnaire in his/her class. Few upper secondary schools and classes participated because of dense learning and exam schedules at these schools. After contacting the teachers, 1,401 candidates were recruited for the study. Among these participants, 248 did not complete the survey and were excluded. Another 14 participants provided inconsistent responses with regard to age (e.g., “30,000,000 years old”), resulting in a final dataset of 1,139 complete and valid questionnaires for analysis (357 bridge-year school students, 667 vocational school students, and 115 upper secondary school students).

### Questionnaire

The questionnaire was modified from questionnaires that were previously used in similar surveys among university students [2,8]. However, the language was adapted to the age of the study group to facilitate understanding the questions that were asked. The full questionnaire is provided as Supplementary Material online (S1 File). The questionnaire contained 34 main questions that were related to additional questions, depending on the answers given. The questionnaires were completed in approximately 15–20 min. Informed consent was provided electronically before starting the survey.

After providing information on sex, age, school, and class, the participants were first asked whether they had heard of the use of prescription drugs (e.g., Ritalin), recreational drugs (e.g., cannabis and cocaine), or other freely available substances (e.g., coffee and energy drinks) to enhance cognitive function (i.e., to improve or extend learning or enhance attention). The participants were then asked whether they knew other students at their school who had used prescription drugs (e.g., Ritalin) without having a medical indication or recreational drugs (e.g., cannabis and cocaine) to improve brain function while studying.

A series of questions were asked about performance pressure and stress. The participants rated the degree of performance pressure they felt with regard to school, apprenticeship, leisure, family, friends/partners on a 4-point Likert scale. The participants were then asked how often they felt stressed during the last 12 months with regard to school, apprenticeship, leisure, family, friends/partners on a 5-point Likert scale. The participants were further asked to indicate reasons for perceiving stress at school during the last 12 months. Additionally, perceived stress during the last 12 months was estimated using four items of the Perceived Stress Scale (PSS) [23]: 1. How often have you felt unable to control the important things in your life? 2. How often have you felt confident about your ability to handle your personal problems? 3. How often have you felt that things were going your way? 4. How often have you felt difficulties were piling up so high that you could not overcome them? Each question was answered on a 5-point Likert scale. Questions 2 and 3 were worded in a positive direction and reverse-scored. The responses were summed to create a psychological stress score that ranged from 0 to 16, with higher scores indicating greater psychological stress. The participants were also asked how important good performance at school was for them and how satisfied they felt with their current life situation.

The participants were then asked whether they had ever used one or more of the following prescription drugs without having a medical indication or recreational drugs or soft enhancers (see below) for performance enhancement while studying (CE) or for recreational purposes. The use of substances to enhance performance while studying or at work (i.e., to perform better during exams, to cope with stress, to improve or extend learning, or to better relax or sleep before a stressful day) was considered CE. Recreational use referred to use at parties to stay awake or dance longer, for fun, or to relax without a specific goal. Prescription drugs included methylphenidate, modafinil, cough medication, activating antidepressants, anti-dementia agents, sedatives/hypnotics, and beta blockers. Both misuse of prescribed drugs (e.g., higher dose, other purpose) and misuse of non-prescribed drugs were included. Dextramphetamine and mixed amphetamine salts are not available as medications in Switzerland and were therefore not included. Recreational drugs included alcohol, cannabis, cocaine, amphetamines, and methylenedioxymethamphetamine (MDMA; ecstasy). Soft enhancers included herbal sedatives (e.g., common valerian), vitamins and tonics (e.g., gingko and vitamin pills), tobacco, coffee, caffeine tablets, and energy drinks. Names of medications including brand names or drugs were provided for each drug class. The prevalence of use was determined for each substance (ever used, last-year use, and last-month use, and the frequency of use during the last month). Additionally, participants indicated the motives for use (e.g., to stay awake/learn longer, to concentrate, to enhance memory, to improve sleep, to relax, to improve grades, to get “high,” etc.), whether their expectations regarding the effects were fulfilled, and whether they would consider the use of the substance again in similar situations.

Attitudes toward CE in school were also explored. Specifically, participants were asked if they agreed with the use of medications and/or psychoactive substances (e.g., cannabis, cocaine, and MDMA) for CE and the reasons that would justify the use for CE in school.

Finally, the students were asked to indicate whether they had ever had a diagnosis of a psychiatric or medical disorder.

### Statistical analyses

The analyses were performed using Statistica 12 software (StatSoft, Tulsa, OK, USA). Proportions were compared using Pearson’s *χ* ^*2*^ test. Differences in Likert scale scores (perceived performance pressure and stress) between students who had used a substance at least once for CE and non-users were analyzed using Wilcoxon rank sum tests. The significance level was *p* < 0.05 without adjusting for multiple tests.

## Results

### Sample description

A total of 1,139 students participated in the study. Participant characteristics are shown in Table 1. The average age was 17.1 years (range: 16–24 years), and most of the participants were 16 years old (*n* = 405, 35.6%) or 17 years old (*n* = 412, 36.2%). None of the upper secondary school students was older than 18 years old. In bridge year schools and vocational-schools, 10 students (0.9%) and 105 students (9.2%), respectively, were ≥ 19 years old. Similar numbers of male and female students in bridge year schools and upper secondary schools participated in the study, whereas significantly more participants from vocational schools were female (Z = 4.16, *p* < 0.001, *n* = 667). Of the total sample (*n* = 1,139), slightly more female than male students participated in the study (Z = 2.97, *p* < 0.01).

**Table 1:** Participant characteristics according to school type.

	Bridge year schools	Vocational schools	Upper secondary schools	total
**Number of participants**	31.3% (357)	58.6% (667)	10.1% (115)	100% (1,139)
**Female**	49.6% (177)	58.0% (387)	48.7% (56)	54.4% (620)
**Male**	50.4% (180)	42.0% (280)	51.3% (59)	45.6% (519)
**Age in years, mean (SD)**	16.7 (0.9)	17.5 (1.4)	16 (0.5)	17.1 (1.3)

### Prevalence of cognitive enhancement, motives for use, and source of supply

The majority of the students (85.9%, 978) reported that they had heard of the use of prescription drugs, recreational drugs, and other substances for CE. Two hundred five participants (18%) were aware of other students in their school who used prescription drugs non-medically for CE, 353 (31%) were aware of other students in their school who used recreational drugs for CE, and 754 (66.2%) reported other people’s use of soft enhancers for CE.

Prevalence rates of substance use for CE or recreational purposes are shown in Table 2. Of the total sample, 621 subjects (54.5%) had used prescription drugs, recreational drugs, and/or soft enhancers at least once for the purpose of CE. Prescription drugs had been used for CE by 105 participants (9.2%), and recreational drugs including alcohol had been used by 71 participants (6.2%; Table 2). Of the total sample, 151 participants (13.3%) had used a prescription drug and/or recreational drug (including alcohol) at least once for CE. The last-year and last-month prevalence rates were lower compared with the lifetime prevalence rates for all of the substance groups (Table 2).

**Table 2:** Prevalence of substance use (N = 1,139).

Purpose of use	Cognitive enhancement			Recreation	Cognitive enhancement and/or recreation
	Ever used	Last-year use	Last-month use	Ever used	Ever used
**Prescription drugs**	**9.2% (105)**	**5.9% (67)**	**4.0% (45)**	**16.9% (192)**	**20.7% (236)**
Methylphenidate	4.0% (46)	2.8% (32)	2.0% (23)	3.5% (40)	6.3% (72)
Cough medication	2.8% (32)	1.4% (16)	1.1% (13)	11.4% (130)	13.2% (150)
Sedatives/hypnotics	2.8% (32)	1.7% (19)	0.9% (10)	5.6% (64)	7.4% (84)
Antidepressants	1.7% (19)	1.3% (15)	1.1% (13)	2.6% (30)	3.2% (37)
Beta blockers	0.7% (8)	0.7% (8)	0.7% (8)	1.0% (12)	1.5% (17)
Modafinil	0.4% (4)	0.3% (3)	0.3% (3)	1.4% (16)	1.6% (18)
Anti-dementia agents	0.4% (5)	0.3% (3)	0.3% (3)	1.2% (14)	1.5% (17)
**Recreational drugs**	**6.2% (71)**	**4.9% (56)**	**4.1% (47)**	**73.4% (836)**	**73.7% (839)**
Cannabis	4.5% (51)	3.4% (39)	3.2% (36)	37.9% (432)	38.3% (436)
Alcohol	1.5% (17)	1.0% (12)	0.6% (7)	71.1% (810)	71.5% (814)
Illegal amphetamines	1.4% (16)	1.0% (12)	1.0% (11)	4.6% (52)	4.9% (56)
Cocaine	1.3% (15)	1.0% (12)	1.0% (11)	3.9% (44)	4.2% (48)
MDMA (ecstasy)	1.1% (13)	0.7% (8)	0.6% (7)	6.2% (71)	6.7% (76)
**Prescription drugs and/or recreational drugs**	**13.3% (151)**	**9.0% (103)**	**6.9% (79)**	**76.4% (870)**	**77.2% (879)**
**Soft enhancers**	**51.3% (584)**	**43.4% (494)**	**37.8% (431)**	**87.1% (992)**	**89.4% (1,018)**
Energy drinks	33.3% (379)	28.4% (323)	24.6% (280)	69.4% (791)	75.4% (859)
Coffee	29.8% (339)	25.1% (286)	21.9% (249)	55.7% (634)	64.3% (732)
Tobacco	12.6% (143)	9.3% (106)	8.3% (95)	52.9% (602)	53.8% (613)
Vitamins and tonics	11.7% (133)	9.0% (103)	6.4% (73)	13.8% (157)	19.8% (225)
Herbal sedatives	8.3% (94)	6.9% (79)	4.3% (49)	10.4% (119)	15.6% (178)
Caffeine tablets	2.5% (28)	1.8% (20)	1.1% (13)	2.5% (29)	4.0% (45)
**Prescription drugs, recreational drugs, and/or soft enhancers (all substances)**	**54.5% (621)**	**45.5% (518)**	**39.5% (450)**	**91.3% (1,040)**	**92.9% (1,058)**

The prescription drug that was most frequently used for CE was methylphenidate (Table 2). Among the participants who reported methylphenidate use for CE during the last month (*n* = 23), eight (34.8%) had used it on more than 20 days, four (17.4%) had used it on 10–19 days, six (26%) had used it on 4–9 days, and five (21.7%) had used it on 1–3 days. Nineteen of the students who reported methylphenidate use for CE (41.3%) noted that their expectations regarding its effects were met, and half of the users (*n* = 24, 52.2%) considered using methylphenidate again in similar situations. One-third of the students who reported prescription drug use for CE (*n* = 35, 33.3%) received the drugs from a physician, 12 (11.4%) received them from other students, nine (8.6%) received them from drug dealers, seven (6.7%) received them from their parents, four (3.8%) received them via the Internet, and three (2.9%) bought them from pharmacies.

The recreational drugs that were most frequently used for CE were cannabis and alcohol, and the use of illegal amphetamines, cocaine, and MDMA was rarely reported (Table 2). Cannabis products most often met the students’ expectations regarding their effects (*n* = 42, 82.4%), and 36 students (70.6%) who used cannabis for CE reported that they would consider future use in similar situations.

The most frequently used soft enhancers for CE were coffee and energy drinks (Table 2). The use of caffeine tablets for CE was not prevalent. However, caffeine tablet users (*n* = 28) were the most satisfied regarding its effects (*n* = 15, 53.6%), and 13 of those students (46.4%) considered using them repeatedly.

Among students who had ever used at least one substance for CE (*n* = 621), the most commonly reported motives for CE were to stay awake (*n* = 613, 98.7%) and improve concentration (*n* = 595, 95.8%). Sixty students (9.7%) who used substances for CE reported such use mainly during exams, 95 (15.3%) reported use most commonly while preparing for exams, and 225 (36.2%) reported use in stressful situations.

Students’ experiences with substance use for CE according to school type are shown in Table 3. Upper secondary school students reported lower CE with prescription drugs and/or recreational drugs compared with bridge year and vocational school students (Table 3).

**Table 3:** Lifetime prevalence of drug use for cognitive enhancement according to substance group and school type.

	Bridge year schools (N = 357)	Vocational schools (N = 667)	Upper secondary schools (N = 115)	total (N = 1,139)
**Prescription drugs**	11.2% (40)	9.0% (60)	4.3% (5)*	9.2% (105)
**Recreational drugs**	7.8% (28)	6.0% (40)	2.6% (3)*	6.2% (71)
**Pescription drugs and/or recreational drugs**	15.4% (55)	13.5% (90)	5.2% (6)**+	13.3% (151)
**Soft enhancers**	40.3% (144)	57.9% (386)***	47.0% (54)	51.3% (584)
**Prescription drugs, recreational drugs, and soft enhancers (all substances)**	44.5% (159)	60.9% (406)***	48.7% (56)+	54.5% (621)
**Non-users**	55.5% (198)	39.1% (261)***	51.3% (59)+	45.5% (518)

*p<0.05

** p<0.01

***p<0.001 compared with bridge year schools

+p<0.05 compared with vocational schools

No significant differences were found between males and females in CE when considering all of the substances together (males: *n* = 268, 51.6%; females: *n* = 353, 56.9%) or when considering prescription drugs only (males: *n* = 50, 9.6%; females: *n* = 55, 8.9%). However, more male students (*n* = 45, 8.7%) reported having used recreational drugs (including alcohol) for CE compared with females (*n* = 26, 4.2%; *χ* ^*2*^ = 9.69, *p* < 0.01). More female students (*n* = 335, 54.0%) reported having used soft enhancers for CE compared with males (*n* = 249, 48.0%; *χ* ^*2*^ = 4.15, *p* < 0.05).

### Attitudes toward cognitive enhancement

Of the total sample, 318 students (27.9%) indicated that they agreed with the use of prescription and/or recreational drugs for CE. More students who used substances for CE (*n* = 197, 31.7%) agreed with the use of substances for CE compared with non-users (*n* = 121, 23.4%; *χ* ^*2*^ = 10.52, *p* < 0.01). Five hundred thirteen students (45.0%) noted that they see no justifiable reasons for CE in schools, whereas 271 (23.8%) reported that CE was acceptable in schools if it was based on a physicians’ advice or in the context of a psychiatric disorder (*n* = 255, 22.4%).

### Stress and performance pressure

Students who perceived more performance pressure at school (score: 3–4, *n* = 579) were more likely to have used at least one substance for CE (*n* = 337, 58.2%) compared with students who perceived no or only minimal performance pressure at school (score: 1–2, *n* = 560, of which 281 [50.2%] used substances for CE; *χ* ^*2*^ = 7.39, *p* < 0.01). Students who had used a substance at least once for CE perceived more performance pressure with regard to school, apprenticeship, family, and friends/partners but not leisure compared with non-users (Table 4). Furthermore, students who felt stressed at school during the last 12 months (score: > 3, *n* = 532) were more likely to have used a substance for CE (*n* = 328, 61.7%) compared with students who felt no or minimal stress (score: < 3, *n* = 243, of which 108 [44.4%] used substances for CE; *χ* ^*2*^ = 20.12, *p* < 0.001). Students who used substances for CE perceived more stress during the last 12 months with regard to school, apprenticeship, leisure, family, and friends/partners compared with non-users (Table 4). Students who used substances for CE had slightly higher scores on the PSS (mean ± SD: 7.6 ± 2.7) compared with non-users (mean ± SD: 7.0 ± 2.9; Z = 3.34, *p* < 0.001). Frequently reported reasons for stress with regard to school included exams (*n* = 791, 69.4%), performance requirements (*n* = 501, 44.0%), and essays/presentations (*n* = 394, 34.6%). Students who had used a substance at least once for CE felt slightly less satisfied with their current life situation compared with non-users (Z = 2.33, *p* < 0.05). Students who used substances for CE and non-users did not differ in the degree to which they considered good performance was important in school (Table 4).

**Table 4:** Perceived performance pressure and stress among students who report the use of prescription drugs, recreational drugs, and/or soft enhancers for cognitive enhancement, and non-users

	Cognitive enhancement users (n = 621)					Non-users (N = 518)					Wilcoxon test
	% (n)					% (n)					Z =
**Performance pressure (score: 1–4)** ^**1**^	**1**	**2**	**3**	**4**		**1**	**2**	**3**	**4**		
School	5.8 (36)	39.6 (246)	41.2 (256)	13.4 (83)		11.8 (61)	41.9 (217)	37.5 (194)	8.9 (46)		3.66; p<0.001
Apprenticeship	15.6 (97)	28.2 (175)	38.2 (237)	18.0 (112)		25.3 (131)	34.7 (180)	27.4 (142)	12.5 (65)		5.44; p<0.001
Leisure	49.3 (306)	30.8 (191)	14.2 (88)	5.8 (36)		53.3 (276)	30.7 (159)	11.6 (60)	4.4 (23)		NS
Familiy	35.4 (220)	42.5 (264)	15.8 (98)	6.3 (39)		45.4 (235)	36.3 (188)	11.4 (59)	6.9 (36)		3.16; p<0.01
Friends/partner	44.9 (279)	34.1 (212)	16.6 (103)	4.3 (27)		55.0 (285)	31.9 (165)	7.9 (41)	5.2 (27)		3.66; p<0.001
**Perceived stress during last 12 months (score: 1–5)** ^**2**^	**1**	**2**	**3**	**4**	**5**	**1**	**2**	**3**	**4**	**5**	
School	4.3 (27)	13.0 (81)	29.8 (185)	33.3 (207)	19.5 (121)	7.1 (37)	18.9 (98)	34.6 (179)	24.9 (129)	14.5 (75)	4.62; p<0.001
Apprenticeship	19.5 (121)	13.0 (81)	23.0 (143)	26.9 (167)	17.6 (109)	31.9 (165)	13.1 (68)	24.9 (129)	18.7 (97)	11.4 (59)	5.47; p<0.001
Leisure	38.2 (237)	33.0 (205)	20.9 (130)	6.4 (40)	1.4 (9)	47.5 (246)	32.2 (167)	15.1 (78)	3.9 (20)	1.4 (7)	3.65; p<0.001
Familiy	20.3 (126)	32.2 (200)	25.9 (161)	12.6 (78)	9.0 (56)	33.4 (173)	29.9 (155)	21.0 (109)	10.0 (52)	5.6 (29)	4.86; p<0.001
Friends/partner	29.1 (181)	32.7 (203)	24.5 (152)	9.0 (56)	4.7 (29)	44.2 (229)	30.9 (160)	17.6 (91)	5.0 (26)	2.3 (12)	5.74; p<0.001
	**1**	**2**	**3**	**4**		**1**	**2**	**3**	**4**		
**Importance of good performance at school (score: 1–4)** ^**3**^	1.8 (11)	5.2 (32)	54.4 (338)	38.6 (240)		2.3 (12)	4.8 (25)	53.5 (277)	39.4 (204)		NS
**Satisfaction with current life situation (score: 1–4)** ^**4**^	6.4 (40)	26.1 (162)	54.6 (339)	12.9 (80)		9.3 (48)	18.3 (95)	52.9 (274)	19.5 (101)		-2.33; p<0.02

^1^1 = not at all, 2 = little, 3 = rather much, 4 = very much

^2^1 = never, 2 = rarely, 3 = sometimes, 4 = often 5 = very often

^3^1 = not at all important, 2 = not important, 3 = important, 4 = very important

^4^1 = very unsatisfied, 2 = rather unsatisfied, 3 = satisfied, 4 = very satisfied

### Psychiatric disorders

Two hundred six students (18.0%) had or have had a diagnosis of a psychiatric disorder. Eighty students (7.0%) reported being diagnosed with depression, 78 (6.8%) reported being diagnosed with attention-deficit disorder (ADD) and/or attention-deficit/hyperactivity disorder (ADHD), 34 (3.0%) reported being diagnosed with an anxiety disorder, 21 (1.8%) reported being diagnosed with a personality disorder, 13 (1.1%) reported being diagnosed with posttraumatic stress disorder, 13 (1.1%) reported being diagnosed with addiction, 11 (1%) reported being diagnosed with obsessive-compulsive disorder, and 35 (3.1%) reported being diagnosed with other psychiatric disorders. Among the participants with a psychiatric disorder (*n* = 206), 139 (67.5%) used substances for CE. Among the participants without a psychiatric disorder (*n* = 933), CE was reported by 479 (51.3%; *χ* ^*2*^ = 17.70, *p* < 0.001). Fifty-four participants (69.2%) with ADD/ADHD (*n* = 78) used substances for CE, and 564 participants (53.2%) without ADD/ADHD (*n* = 1,061) used substances for CE (*χ* ^*2*^ = 7.56, *p* < 0.01).

## Discussion

The present study investigated the prevalence and motives for CE among school students in the Canton of Zurich, Switzerland. The main finding was that a significant proportion of Swiss school students (54.5%) reported having used substances at least once for CE at school. This high prevalence resulted from using a very wide definition of CE, which included not only prescription and recreational drugs but also commonly used and freely available soft enhancers in comparison to studies which considered only the use of medications without soft enhancers as CE [22]. Students’ substance use for CE was most often linked to the use of soft enhancers, mainly energy drinks and coffee. Consistent with previous studies, methylphenidate was the prescription drug that was most commonly used for CE. The use of cannabis and alcohol not only recreationally but also for direct or indirect CE was reported by few students. However, after excluding soft enhancers, we still found that 13.3% of the students had used prescription or recreational drugs at least once for CE. Substances were used for CE mainly to stay awake and improve concentration. Male students were more familiar with recreational drug use for CE, and female students had higher rates of soft enhancer use. In particular, students with CE experience felt more performance pressure and stress. A previous diagnosis of a psychiatric disorder, such as ADD/ADHD, was also associated with higher rates of substance use for CE.

The lifetime prevalence rate of 13.3% for CE with prescription and recreational drugs (including alcohol) in the present study was similar to the 13.8% rate that was reported among Swiss university students [2]. However, the rate of substance use for CE was higher among Swiss university students than among school students when all substance use (including soft enhancers) was considered (71.4% *vs*. 54.5%, respectively).

The present findings demonstrate that substance use for CE is prevalent among younger students at the secondary school level before they enter institutions of higher education. This means that any preventive actions including providing information on risks associated with CE should be taken already at the secondary school level. This is the first study providing information on CE use prevalence among school students in Switzerland. It is important to know what substances are actually consumed for CE and what the use prevalence rates are to further investigate risks of CE use in future studies. Additionally, we showed an association between perceived performance pressure and stress at school and CE use. Assuming that stressed students are more likely to perform CE, providing alternative coping strategies could be part of future preventive measures.

Compared with data from school students in Germany [22], the lifetime prevalence for prescription and illicit drug use for CE was higher in the present study (9.2% *vs*. 1.6% and 6.2% *vs*. 2.4%, respectively). However, the German study only investigated the use of some prescription and illegal stimulants for CE (i.e., methylphenidate, modafinil, amphetamines, cocaine, and ecstasy), whereas the present study examined the use of additional substances, including sedative drugs, for indirect CE. The last-year and last-month prevalence rates for CE were significantly lower than lifetime use rates in all studies, indicating that CE rarely occurs on a regular basis.

Consistent with other studies, male students were more experienced with the use of recreational drugs for CE, and female students were more experienced with the use of soft enhancers for CE [2,8,24]. Moreover, students who had used a substance at least once for CE felt stressed more often compared with non-users [8]. However, no casual relationship can be identified between stress and CE based on the results of a cross-sectional study. Possible explanations for the higher lifetime prevalence of CE in vocational school students could be that these students were older and received a salary during the apprenticeship.

The higher prevalence of prescription drug use compared with recreational drugs including alcohol in the present study could indicate easier access to those substances (i.e., drug prescriptions for other indications and over-the-counter cough medications) or may be related to drugs of abuse (including alcohol) that are used mostly in recreational settings and not for CE. Another interesting finding in the present study was that in some cases (e.g., methylphenidate) the number of students who considered repeated substance use for CE was higher than the number of students who reported that such use met their expectations after use. Thus, some students considered repeated substance use for CE but were not satisfied with the performance results. Data on the efficacy of different substances to enhance cognition are relatively inconsistent and could explain differences between students’ perceptions of whether such use meets expectations [1,3,10,21,25–27]. It is unlikely that everyone will benefit equally from CE. A recent study suggested that regular users of substances for CE present a personality profile that might explain substance use and efficacy [28].

In many previous studies [21,29], individuals with an ADD/ADHD or other psychiatric diagnosis that required medication were excluded to prevent a false prevalence assessment of drug use for CE. However, an estimated 25% of patients who are diagnosed with ADD/ADHD misuse their prescribed medication [6,30]. The misuse of a prescribed drug to enhance cognitive performance would represent CE, and such a situation, therefore, would be included in the present study. Similar to other studies [30,31], our results showed that students with ADD/ADHD had significantly more experience with CE, and methylphenidate was the most prevalent prescription drug that was used for this purpose. This is, however, also a limitation of the present study because one possibility is that some students had overlooked the term “nonmedical use” that was described in the introduction to the questionnaire (and not repeated in the individual questions) and thus reported the use of their methylphenidate prescription as CE, in which coping with their ADD/ADHD symptoms also helps with school performance. Additionally, all eight participants who reported methylphenidate use on more than 20 days during the last month had a diagnosis of ADD/ADHD; therefore, their drug use for CE might still be prescribed.

The present study has some additional limitations that should be considered for the interpretation of the findings. First, the study did not include students from all of the schools in the Canton of Zurich because study participation was denied by school administrations in some cases. This was mainly attributable to other student surveys that were being conducted, the ongoing Bologna reform evaluation in some upper secondary schools, and time constraints. Although all answers were tested for their plausibility before inclusion, some of the participants may not have answered all of the questions honestly, or they may not have understood some of the questions or terms. Furthermore, some of the students might have tried to provide socially acceptable answers to questions on sensitive issues, such as illegal drug use, which would result in an underestimation of the prevalence rates. However, the similar results in the previous study among Swiss university students [2] indicate the validity of the present results and are still quite high for the present younger study population.

Another possible limitation is that the study was conducted during a quiet exam-free school period (before and after vacation) to avoid being a burden for the students and teachers, although stressful periods are of greater interest for the study question. Nevertheless, the last-month prevalence of substance use for CE would be the only result that might have changed if the survey was administered parallel to exams. In such a case, however, we might have lost students’ interest in the survey and even valid data if the survey was answered too quickly because of a lack of time or not at all because of upcoming exams.

The minimum age of the participants was 16 years, thus significantly reducing the study population, which is another limitation. The number of participants from upper secondary schools was rather small compared with the other two school types. Moreover, all of the participants lived in the Canton of Zurich, and the results may not be generalizable to other Swiss schools. Finally, our findings are limited by the use of the cross-sectional design.

## Conclusions

Half of the students in the Canton of Zurich in the present study had used a substance at least once to improve school performance. Readily available soft enhancers were the substances that were most commonly used. The prevalence rates for the use of prescription and recreational drugs for CE were similar to those reported by Swiss university students, indicating that CE should be addressed before students enter systems of higher education. Performance pressure, stress, and psychiatric disorders appear to be associated with substance use for CE and need to be considered when planning preventive interventions.

## Supporting Information

S1 File Questionnaire. (PDF)
