# Supplementary material for: The Use of Prescription Drugs, Recreational Drugs, and “Soft Enhancers” for Cognitive Enhancement among Swiss Secondary School Students
Source: PLoS One. 2015 Oct 27;10(10):e0141289. doi: 10.1371/journal.pone.0141289 (PMC4624689; doi:10.1371/journal.pone.0141289)
Supplement: S1 File — (PDF) [file pone.0141289.s001.pdf]

Stress und Hirndoping an Berufsvorbereitungs-, Berufsfach- und Mittelschulen im Kanton Zürich

Eine Studie des Schweizer Instituts für Sucht- und Gesundheitsforschung

Liebe Schülerin, lieber Schüler

In den Medien ist in letzter Zeit immer wieder von „Hirndoping" die Rede. Unter Hirndoping versteht man die Einnahme von Medikamenten und/oder anderen Substanzen mit dem Ziel die geistige Leistungsfähigkeit zu verbessern (zum Beispiel um länger lernen zu können, sich besser in der Schule zu konzentrieren oder die Nervosität vor oder während einer Prüfung zu reduzieren). Laut Ergebnissen aus der Forschung greifen manche Schüler und Studierende, besonders während anspruchsvollen und stressigen Zeiten, wie vor Prüfungen, zu diesen „Hilfsmitteln".
Wir führen deshalb eine anonyme Befragung zur aktuellen Situation von Schülerinnen und Schülern an Berufsfach-, Berufsvorbereitungs- und Mittelschulen des Kantons Zürich, zum Umgang mit Stress in der Schule und im speziellen zu Hirndoping durch.
Die Beantwortung dieses Fragebogens dauert ca. 15 Minuten.
Du hast jederzeit die Möglichkeit deine Teilnahme vorzeitig ohne Angaben von Gründen abzubrechen. Wir bitten Dich aber den Fragebogen möglichst vollständig und möglichst wahrheitsgetreu zu beantworten.
Die Befragung erfolgt anonym, das heisst es kann kein Rückschluss auf deine Person und kein Zusammenhang zu deinen Antworten hergestellt werden. Nach Abschluss des Fragebogens werden die Daten mit einem Sicherheitsschlüssel an einen Sicherheitsserver der Universität Zürich übermittelt.

Mit der Teilnahme an dieser Befragung leistest Du einen wichtigen Beitrag für die Gesundheitsforschung in der Schweiz.

Bitte lies die Fragen genau durch und beantworte sie ehrlich.

Herzlichen Dank für Deine Teilnahme.

Projektleitung:
PD Dr. Michael Schaub, Direktor Schweizer Institut für Sucht- und Gesundheitsforschung; lic. phil Domenic Schnoz, Leiter Zürcher Fachstelle für Alkohol- und Medikamentenprobleme; Vigeli Venzin, Leiter Fachstelle Suchtprävention Mittelschulen und Berufsbildung

Einverständniserklärung

1
Einverständniserklärung zur Befragung: „Stress und Hirndoping an Berufsvorbereitungs-, Berufsfach- und Mittelschulen des Kantons Zürich“

Ich bestätige, dass ich freiwillig an der oben genannten Befragung teilnehme. Ich werde in dieser Befragung am Computer Angaben zu meiner Person (anonymisiert), meinem Umgang mit Stress und zu Hirndoping machen.
Die Befragung dauert etwa 15 Minuten.

Ich bin einverstanden, dass meine Befragungsangaben in vollständig anonymisierten Form, so dass keine Rückschlüsse auf meine Person gemacht werden können, an das Schweizer Institut für Sucht- und Gesundheitsforschung der Universität Zürich weiter geleitet, dort ausgewertet und über die Gesamtergebnisse berichtet werden darf.

Ich habe Kenntnis davon genommen, dass ich jederzeit die Möglichkeit habe die Befragung ohne Angaben von Gründen zu beenden und dass sich keine Nachteile durch eine Nicht-Teilnahme an der Studie ergeben.

Bitte wählen Sie alle zutreffenden Antworten aus:

- ☐ Ich habe den Text der Einverständniserklärung gelesen, verstanden und bin damit einverstanden

Solltest Du im Nachhinein Fragen haben, darfst Du dich gerne an uns wenden (hirndopingfragen@hotmail.com).

Schule und Beruf

Ein paar Fragen zu deiner schulischen und/oder beruflichen Situation.

2 Welche Schule besuchst Du?
Bitte wählen Sie nur eine der folgenden Antworten aus:

○ Berufsfachschule/Lehre
○ Mittelschule/Gymnasium
○ Berufsvorbereitungsschule/10. Schuljahr

3 Welches Berufsschuljahr/Lehrjahr besuchst Du?
Beantworten Sie diese Frage nur, wenn folgende Bedingungen erfüllt sind:
Antwort war 'Berufsfachschule/Lehre' bei Frage 2 (Welche Schule besuchst Du?)
Bitte wählen Sie nur eine der folgenden Antworten aus:

○ Erstes Jahr
○ Zweites Jahr
○ Drittes Jahr
○ Viertes Jahr

4 Welche Berufsfachschule besuchst Du?
Beantworten Sie diese Frage nur, wenn folgende Bedingungen erfüllt sind:
Antwort war 'Berufsfachschule/Lehre' bei Frage 2 (Welche Schule besuchst Du?)
Bitte wählen Sie nur eine der folgenden Antworten aus:

○ Allgemeine Berufsschule Zürich (ABZ)
○ Baugewerbliche Berufsschule Zürich (BBZ)

○ Berufsbildungsschule Winterthur (BBW)
○ Berufsbildungszentrum Dietikon (BZD)
○ Berufsfachschule Winterthur (BFS)
○ Berufsschule Bülach (BSB)
○ Berufsschule für Detailhandel Zürich (DHZ)
○ Berufsschule für Gestaltung Zürich (BGZ)
○ Berufsschule für Hörgeschädigte Zürich (BSFH)
○ Berufsschule Mode und Gestaltung Zürich (MGZ)
○ Berufsschule Rüti (BSR)
○ Gewerblich-Industrielle Berufsschule Uster (GIBU)
○ Bildungszentrum Zürichsee (BZZ)
○ Careum Bildungszentrum (CBZ)
○ Gewerbliche Berufsschule Wetzikon (GBW)
○ KV Zürich Business School (KVZBS)
○ Landwirtschaftliches Bildungszentrum Strickhof
○ Modeco
○ MSW-Winterthur
○ Technische Berufsschule Zürich (TBZ)
○ Wirtschaftsschule KV Uster (WSKVU)
○ Wirtschaftsschule KV Wetzikon (WKVW)
○ Wirtschaftsschule Winterthur (WSKVW)
○ Zentrum für Ausbildung im Gesundheitswesen (ZAG)
○ Sonstiges

5 Welche Lehre machst Du?
Beantworten Sie diese Frage nur, wenn folgende Bedingungen erfüllt sind:
Antwort war 'Berufsfachschule/Lehre' bei Frage 2 (Welche Schule besuchst Du?)
Bitte wählen Sie nur eine der folgenden Antworten aus:

○ Agrarpraktiker/in EBA
○ Anlagen- und Apparatebauer/in EFZ
○ Anlagenführer/in EFZ
○ Architekturmodellbauer/in (EFZ)
○ Assistent/in Gesundheit und Soziales EBA
○ Augenoptiker/in EFZ
○ Automatiker/in EFZ
○ Automatikmonteur/in EFZ
○ Automobil-Assistent/in EBA
○ Automobil-Fachmann/-frau EFZ
○ Automobil-Mechatroniker/in EFZ
○ Bäcker/in-Konditor/in-Confiseur/in EBA

- ◯ Bäcker/in-Konditor/in-Confiseur/in EFZ
- ◯ Baumaschinenmechaniker/in EFZ
- ◯ Baupraktiker/in EBA
- ◯ Bauwerktrenner/in EFZ
- ◯ Bekleidungsgestalter/in EFZ
- ◯ Betonwerker/in EFZ
- ◯ Boden-Parkettleger/in EFZ
- ◯ Bootbauer/in (EFZ)
- ◯ Buchhändler/in EFZ
- ◯ Bühnentänzer/in EFZ
- ◯ Büroassistent/in EBA
- ◯ Carrossier/in Lackiererei EFZ
- ◯ Carrossier/in Spenglerei EFZ
- ◯ Coiffeur/-euse EBA
- ◯ Coiffeur/-euse EFZ
- ◯ Dentalassistent/in EFZ
- ◯ Detailhandelsassistent/in EBA
- ◯ Detailhandelsfachmann/-frau EFZ, Schwerpunkt Beratung
- ◯ Detailhandelsfachmann/-frau EFZ, Schwerpunkt Bewirtschaftung
- ◯ Diätkoch/-köchin EFZ
- ◯ Drogist/in EFZ
- ◯ Drucktechnologe/-login EFZ
- ◯ Elektroinstallateur/in EFZ
- ◯ Elektroniker/in EFZ
- ◯ Elektroplaner/in EFZ
- ◯ Entwässerungstechnologe/-login EFZ
- ◯ Fachmann/-frau Betreuung EFZ
- ◯ Fachmann/-frau Betriebsunterhalt EFZ
- ◯ Fachmann/-frau Bewegungs- und Gesundheitsförderung EFZ
- ◯ Fachmann/-frau Gesundheit EFZ
- ◯ Fachmann/-frau Hauswirtschaft EFZ
- ◯ Fachmann/-frau Information und Dokumentation EFZ
- ◯ Fachmann/-frau Kundendialog EFZ
- ◯ Fahrradmechaniker/in EFZ
- ◯ Fahrzeugschlosser/in EFZ
- ◯ Fleischfachassistent/in EBA
- ◯ Fleischfachmann/-frau EFZ
- ◯ Flexodrucker/in EFZ
- ◯ Florist/in EFZ

- Gärtner/in EBA
- Gärtner/in EFZ
- Gebäudereiniger/in EBA
- Gebäudereiniger/in EFZ
- Gebäudetechnikplaner/in Heizung EFZ
- Gebäudetechnikplaner/in Lüftung EFZ
- Gebäudetechnikplaner/in Sanitär EFZ
- Gemüsegärtner/in EFZ
- Gestalter/in Werbetechnik EFZ
- Gipser/in (EFZ)
- Glaser/in EFZ
- Gleisbauer/in EFZ
- Gleisbaupraktiker/in EBA
- Grafiker/in EFZ
- Grundbauer/in EFZ
- Gussformer/in EFZ
- Gusstechnologe/-login EFZ
- Haustechnikpraktiker/in EBA
- Hauswirtschaftspraktiker/in EBA
- Heizungsinstallateur/in EFZ
- Holzbearbeiter/in EBA
- Holzhandwerker/in EFZ
- Hotelfachmann/-frau EFZ
- Hotellerieangestellte/r EBA
- Industrie- und Unterlagsbodenbauer/in EFZ
- Industrie- und Unterlagsbodenbaupraktiker/in EBA
- Industrielackierer/in EFZ
- Informatiker/in EFZ
- Informatikpraktiker/in EBA
- Innendekorateur/in (EFZ)
- Isolierspengler/in EFZ
- Kältesystem-Monteur/in EFZ
- Kaminfeger/in EFZ
- Kaufmann/-frau EFZ, Basis-Grundbildung
- Kaufmann/-frau EFZ, erw. Grundbildung
- Keramiker/in EFZ
- Kleinmotorrad- und Fahrradmechaniker/in EFZ
- Koch/Köchin EFZ
- Konstrukteur/in EFZ

○ Kosmetiker/in EFZ
○ Küchenangestellte/r EBA
○ Kunststofftechnologe/-login EFZ
○ Laborant/in EFZ
○ Landmaschinenmechaniker/in EFZ
○ Landwirt/in EFZ
○ Lebensmittelpraktiker/in EBA
○ Lebensmitteltechnologe/-login EFZ
○ Logistiker/in EBA
○ Logistiker/in EFZ
○ Maler/in (EFZ)
○ Maurer/in EFZ
○ Mechanikpraktiker/in EBA
○ Mediamatiker/in EFZ
○ Medizinische/r Praxisassistent/in EFZ
○ Metallbauer/in EFZ
○ Metallbaukonstrukteur/in EFZ
○ Metallbaupraktiker/in EBA
○ Metalldrücker/in (EFZ)
○ Milchpraktiker/in EBA
○ Milchtechnologe/-login EFZ
○ Montage-Elektriker/in EFZ
○ Motorgerätemechaniker/in EFZ
○ Motorradmechaniker/in EFZ
○ Müller/in EFZ
○ Multimediaelektroniker/in EFZ
○ Netzelektriker/in EFZ
○ Oberflächenbeschichter/in EFZ
○ Obstfachmann/-frau EFZ
○ Ofenbauer/in EFZ
○ Orthopädieschuhmacher/in EFZ
○ Pferdefachmann/-frau EFZ
○ Pferdewart/in EBA
○ Pflästerer/in EFZ
○ Pharma-Assistent/in EFZ
○ Plattenleger/in EFZ
○ Plattenlegerpraktiker/in EBA
○ Podologe/-in EFZ
○ Polybauer/in EFZ

○ Polybaupraktiker/in EBA
○ Polygraf/in EFZ
○ Polymechaniker/in EFZ
○ Printmedienverarbeiter/in EFZ
○ Produktionsmechaniker/in EFZ
○ Recyclist/in EFZ
○ Reifenpraktiker/in EBA
○ Restaurationsangestellte/r EBA
○ Restaurationsfachmann/-frau EFZ
○ Säger/in Holzindustrie EFZ
○ Sanitärinstallateur/in EFZ
○ Schreiner/in EFZ
○ Schreinerpraktiker/in EBA
○ Schuhmacher/in EFZ
○ Spengler/in EFZ
○ Steinbildhauer/in EFZ
○ Steinmetz/in EFZ
○ Strassenbauer/in EFZ
○ Strassenbaupraktiker/in EBA
○ Strassentransportfachmann/-frau EFZ
○ Systemgastronomiefachmann/-frau EFZ
○ Telematiker/in EFZ
○ Textilpfleger/in EFZ
○ Tiermedizinische/r Praxisassistent/in EFZ
○ Tierpfleger/in EFZ
○ Winzer/in EFZ
○ Zahntechniker/in EFZ
○ Zeichner/in EFZ
○ Zimmermann/Zimmerin EFZ
○ Sonstiges

6 Welche Klasse besucht du?
Beantworten Sie diese Frage nur, wenn folgende Bedingungen erfüllt sind:
Antwort war 'Mittelschule/Gymnasium' bei Frage 2 (Welche Schule besuchst Du?)
Bitte wählen Sie nur eine der folgenden Antworten aus:

○ 1. Klasse Langzeitgymnasium
○ 2. Klasse Langzeitgymnasium
○ 3. Klasse Langzeitgymnasium/1. Klasse Kurzzeitgymnasium
○ 4. Klasse Langzeitgymnasium/2. Klasse Kurzzeitgymnasium
○ 5. Klasse Langzeitgymnasium/3. Klasse Kurzzeitgymnasium

○ 6. Klasse Langzeitgymnasium/4. Klasse Kurzzeitgymnasium

7 Welche Kantonsschule/Gymnasium besuchst Du?
Beantworten Sie diese Frage nur, wenn folgende Bedingungen erfüllt sind:
Antwort war 'Mittelschule/Gymnasium' bei Frage 2 (Welche Schule besuchst Du?)
Bitte wählen Sie nur eine der folgenden Antworten aus:

○ Kantonsschule Büelrain, Winterthur (KBW)

○ Kantonsschule Enge (KEN)

○ Kantonsschule Freudenberg (KFR)

○ Kantonsschule Uster (KGL)

○ Kantonsschule Hohe Promenade (KSHP)

○ Kantonsschule Hottingen (KSH)

○ Kantonsschule Im Lee (KLW)

○ Kantonsschule Küsnacht (KSK)

○ Kantonsschule Limmattal (KSL)

○ Kantonsschule Zürich Nord (KZN)

○ Kantonsschule Rychenberg (KRW)

○ Kantonsschule Stadelhofen (KST)

○ Kantonsschule Wiedikon (KWI)

○ Kantonsschule Zürcher Oberland (KZO)

○ Kantonsschule Zürcher Unterland (KZU)

○ Liceo Artistico

○ Literargymnasium Rämibühl (LG)

○ Mathematisch-Naturwissenschaftliches Gymnasium Rämibühl (MNG)

○ Realgymnasium Ramibühl (RG)

○ Sonstiges

8 Welches ist Dein Profil?
Beantworten Sie diese Frage nur, wenn folgende Bedingungen erfüllt sind:
Antwort war 'Mittelschule/Gymnasium' bei Frage 2 (Welche Schule besuchst Du?)
Bitte wählen Sie nur eine der folgenden Antworten aus:

○ Neusprachliches Profil (Englisch, Italienisch, Spanisch)

○ Altsprachliches Profil (Latein, Griechisch)

○ Wirtschaftliches Profil

○ Mathematisch-naturwissenschaftliches Profil

○ Musisches Profil

○ Künstlerisches oder sportliches Profil

9 Welche Berufsvorbereitungsschule besuchst Du?
Beantworten Sie diese Frage nur, wenn folgende Bedingungen erfüllt sind:
Antwort war 'Berufsvorbereitungsschule/10. Schuljahr' bei Frage 2 (Welche Schule besuchst Du?)
Bitte wählen Sie nur eine der folgenden Antworten aus:

○ Berufswahlschule Bülach

○ Berufswahlschule Effretikon

○ Berufswahlschule Bezirk Horgen

○ Berufswahlschule Kloten

○ Tempus – Berufsvorbereitung und Erwachsenenbildung am See, Küssnacht

○ Berufswahlschule Limmattal

○ Berufswahlschule Uster

○ Berufswahl- und Weiterbildungsschule Zürcher Oberland, Wetzikon

○ Berufswahlschule Winterthur

○ Werkjahrschule Winterthur

○ Hauswirtschaftliche Fortbildungsschule Winterthur

○ Fachschule Viventa Zürich

○ Sonstiges

10 Welches ist Dein Profil?
Beantworten Sie diese Frage nur, wenn folgende Bedingungen erfüllt sind:
Antwort war 'Berufsvorbereitungsschule/10. Schuljahr' bei Frage 2 (Welche Schule besuchst Du?)
Bitte wählen Sie nur eine der folgenden Antworten aus:

○ BVJ Berufswahl Profil A

○ BVJ Berufswahl Profil B

○ Berufs(feld)orientierter Angebotstyp: BVJ Berufsfeld

○ Berufs(feld)orientierter Angebotstyp: BVJ Vorlehre

○ Integrationsorientierter Angebotstyp: BVJ Sprache und Kultur

Demographische Angaben

11 Geschlecht
Bitte wählen Sie nur eine der folgenden Antworten aus:

○ Weiblich

○ Männlich

12 Wie alt bist Du?

In dieses Feld dürfen nur Zahlen eingegeben werden.

Bitte geben Sie Ihre Antwort hier ein:

13 In welchem Bezirk des Kantons Zürich wohnst Du?
Bitte wählen Sie nur eine der folgenden Antworten aus:

○ Bezirk Affoltern

○ Bezirk Andelfingen

○ Bezirk Bülach

○ Bezirk Dielsdorf

○ Bezirk Dietikon

○ Bezirk Hinwil

○ Bezirk Horgen

○ Bezirk Meilen

○ Bezirk Pfäffikon

○ Bezirk Uster

○ Bezirk Winterthur

○ Bezirk Zürich

○ Sonstiges

14 Welchen Schulabschluss hat dein Vater?
Bitte wählen Sie nur eine der folgenden Antworten aus:

○ keinen Schulabschluss

○ Grundschule/Primarschule

○ Abschluss an einer Sonderschule/Förderschule

○ Sekundarabschluss

○ Matura

○ sonstiger Schulabschluss und zwar:

○ Ich weiss es nicht

○ Sonstiges

15 Welchen Schulabschluss hat deine Mutter?
Bitte wählen Sie nur eine der folgenden Antworten aus:

○ keinen Schulabschluss

○ Grundschule/Primarschule

○ Abschluss an einer Sonderschule/Förderschule

○ Sekundarabschluss

○ Matura

○ sonstiger Schulabschluss und zwar:

○ Ich weiss es nicht

○ Sonstiges

16 Welche berufliche Ausbildung hat dein Vater?
Bitte wählen Sie nur eine der folgenden Antworten aus:

○ Keine abgeschlossene Ausbildung

○ Abgeschlossene Lehre

○ Fachhochschulabschluss

○ Hochschulabschluss (Universitätsabschluss)

○ Promotion (Doktorprüfung)

○ Ich weiss es nicht

○ Sonstiges

17 Welche berufliche Ausbildung hat deine Mutter?

Bitte wählen Sie nur eine der folgenden Antworten aus:

- Keine abgeschlossene Ausbildung
- Abgeschlossene Lehre
- Fachhochschulabschluss
- Hochschulabschluss (Universitätsabschluss)
- Promotion (Doktorprüfung)
- Ich weiss es nicht
- Sonstiges

18 Welchen Beruf übt Dein Vater momentan aus?
Bitte geben Sie Ihre Antwort hier ein:

19 Welchen Beruf übt Deine Mutter momentan aus?
Bitte geben Sie Ihre Antwort hier ein:

Einführung

20 Hast Du schon einmal davon gehört, dass man rezeptpflichtige Medikamente (wie zum Beispiel Ritalin®), psychoaktive Substanzen (wie zum Beispiel Cannabis, Kokain, Ecstasy etc.) und/oder andere Mittel (wie zum Beispiel Kaffee, Energy Drinks, Kaffee, Tabak etc.) konsumieren kann, um die Gehirnleistung zu verbessern (zum Beispiel um besser oder länger zu lernen, die Aufmerksamkeit zu steigern etc.)?
Bitte wählen Sie nur eine der folgenden Antworten aus:

- Ja
- Nein

21 Kennst Du Personen in deiner Schule, die rezeptpflichtige Medikamente (wie zum Beispiel *Ritalin*®) ohne medizinischen Grund einnehmen, um ihre Gehirnleistung für die Schule zu verbessern?
Beantworten Sie diese Frage nur, wenn folgende Bedingungen erfüllt sind:
Antwort war 'Ja' bei Frage 20 (Hast Du schon einmal davon gehört, dass man rezeptpflichtige Medikamente (wie zum Beispiel Ritalin®), psychoaktive Substanzen (wie zum Beispiel Cannabis, Kokain, Ecstasy etc.) und/oder andere Mittel (wie zum Beispiel Kaffee, Energy Drinks, Kaffee, Tabak etc.) konsumieren kann, um die Gehirnleistung zu verbessern (zum Beispiel um besser oder länger zu lernen, die Aufmerksamkeit zu steigern etc.)?)
Bitte wählen Sie nur eine der folgenden Antworten aus:

- Ja
- Nein

22 Kennst Du Personen in deiner Schule, die psychoaktive Substanzen (wie zum Beispiel *Cannabis, Kokain, Ecstasy* etc.) konsumieren, um ihre Gehirnleistung für die Schule zu verbessern?
Bitte wählen Sie nur eine der folgenden Antworten aus:

- Ja
- Nein

23 Kennst Du Personen in deiner Schule, die sonstige Mittel (wie zum Beispiel *Kaffee, Energy Drinks, Tabak* etc.) konsumieren, um ihre Gehirnleistung für die Schule zu verbessern?
Bitte wählen Sie nur eine der folgenden Antworten aus:

- Ja

◯ Nein

Leistungsdruck und Stress

24 Wie stark verspürst Du in den folgenden Bereichen Leistungsdruck?
Bitte wählen Sie die zutreffende Antwort für jeden Punkt aus:

	überhaupt nicht	ein wenig	ziemlich stark	sehr stark
Schule	◯	◯	◯	◯
Lehre	◯	◯	◯	◯
Freizeit/ Hobby	◯	◯	◯	◯
Familie	◯	◯	◯	◯
Freunde und Partner/in	◯	◯	◯	◯

25 Wie häufig hast Du dich in den letzten 12 Monaten gestresst gefühlt?
Bitte wählen Sie nur eine der folgenden Antworten aus:

◯ nie
◯ selten
◯ manchmal
◯ häufig
◯ sehr häufig

26 Wie häufig hast Du dich in den letzten 12 Monaten in folgenden Bereichen gestresst gefühlt?
Beantworten Sie diese Frage nur, wenn folgende Bedingungen erfüllt sind:
Antwort war 'selten ' *oder* 'manchmal ' *oder* 'häufig' *oder* 'sehr häufig' bei Frage 25 (Wie häufig hast Du dich in den letzten 12 Monaten gestresst gefühlt?)
Bitte wählen Sie die zutreffende Antwort für jeden Punkt aus:

	nie	selten	manchmal	häufig	sehr häufig
Schule	◯	◯	◯	◯	◯
Lehre	◯	◯	◯	◯	◯
Freizeit/ Hobby	◯	◯	◯	◯	◯
Familie	◯	◯	◯	◯	◯
Freunde und Partner/in	◯	◯	◯	◯	◯

27 Hast Du dich im Zusammenhang mit Deiner Schule schon einmal während mehreren Wochen gestresst gefühlt? Beachte, dass hier nur nach dem Bereich der Schule gefragt wird und nicht die Lehre gemeint ist.
Beantworten Sie diese Frage nur, wenn folgende Bedingungen erfüllt sind:
Antwort war 'sehr häufig' *oder* 'selten' *oder* 'manchmal' *oder* 'häufig' bei Frage 26 (Wie häufig hast Du dich in den letzten 12 Monaten in folgenden Bereichen gestresst gefühlt? (Schule))
Bitte wählen Sie nur eine der folgenden Antworten aus:

◯ Ja
◯ Nein

28 Hast Du dich im Zusammenhang mit Deiner Lehre (Beruf) schon einmal während mehreren Wochen gestresst gefühlt?
Beantworten Sie diese Frage nur, wenn folgende Bedingungen erfüllt sind:
Antwort war 'Berufsfachschule/Lehre' bei Frage 2 (Welche Schule besuchst Du?) *und* Antwort war 'selten' *oder*

'manchmal' *oder* 'häufig' *oder* 'sehr häufig' bei Frage 26 (Wie häufig hast Du dich in den letzten 12 Monaten in folgenden Bereichen gestresst gefühlt? (Lehre))

Bitte wählen Sie nur eine der folgenden Antworten aus:

- ○ Ja
- ○ Nein

29 Was sind Gründe warum Du dich in den letzten 12 Monaten in der Schule gestresst gefühlt hast?

Bitte wählen Sie alle zutreffenden Antworten aus:

- ☐ Leistungsanforderungen
- ☐ Orientierungsproblem
- ☐ Konkurrenz unter Schüler/innen
- ☐ Gruppendruck
- ☐ Prüfungen
- ☐ Schriftliche Arbeiten und Referate
- ☐ Hausaufgaben
- ☐ Beteiligung an Diskussionen im Unterricht
- ☐ Bewältigung des Stoffumfangs
- ☐ Mich für meine Fächer zu begeistern
- ☐ Schwierigkeiten mit Lehrpersonen
- ☐ Ich habe mich in diesem Bereich nicht gestresst gefühlt
- ☐ Sonstiges:

30 Was sind Gründe warum Du dich in den letzten 12 Monaten in der Lehre gestresst gefühlt hast?

Beantworten Sie diese Frage nur, wenn folgende Bedingungen erfüllt sind:

Antwort war 'Berufsfachschule/Lehre' bei Frage 2 (Welche Schule besuchst Du?)

Bitte wählen Sie alle zutreffenden Antworten aus:

- ☐ Leistungsanforderungen
- ☐ Orientierungsproblem
- ☐ Konkurrenz unter Lernenden
- ☐ Gruppendruck
- ☐ Mich für meine Lehre zu begeistern
- ☐ Schwierigkeiten mit Berufsbildner/-in
- ☐ Ich habe mich in diesem Bereich nicht gestresst gefühlt
- ☐ Sonstiges:

31 Was sind Gründe warum Du dich in den letzten 12 Monaten in privaten Bereichen (Freizeit/Hobby, Familie, Freunde etc.) gestresst gefühlt hast?

Bitte wählen Sie alle zutreffenden Antworten aus:

- ☐ Finanzielle Probleme
- ☐ Eltern
- ☐ Freunde
- ☐ Partnerschaft
- ☐ Aussehen

☐ Ich habe mich in diesem Bereich nicht gestresst gefühlt
☐ Sonstiges:

32 Wie oft ist es Dir möglich ohne den Konsum von Substanzen (zum Beispiel Alkohol, Medikamente etc.) Stress zu bewältigen respektive trotz Stress wieder abschalten zu können?
Bitte wählen Sie nur eine der folgenden Antworten aus:
- ○ nie
- ○ selten
- ○ manchmal
- ○ häufig
- ○ sehr häufig

33 Die folgenden Fragen beschäftigen sich damit, wie häufig Du dich während der letzten 12 Monate durch Stress belastet fühltest.

Bitte wählen Sie die zutreffende Antwort für jeden Punkt aus:

	nie	selten	manchmal	häufig	sehr oft
Wie oft hattest Du in den letzten 12 Monaten das Gefühl, wichtige Dinge in Deinem Leben nicht beeinflussen zu können?	○	○	○	○	○
Wie oft hast Du Dich in den letzten 12 Monaten sicher im Umgang mit Deinen persönlichen Aufgaben und Problemen gefühlt?	○	○	○	○	○
Wie oft hattest Du in den letzten 12 Monaten das Gefühl, dass sich die Dinge nach Deinen Vorstellungen entwickeln?	○	○	○	○	○
Wie oft hattest Du in den letzten 12 Monaten das Gefühl, dass sich Aufgaben oder Probleme so sehr aufgestaut haben, dass Du diese nicht bewältigen kannst?	○	○	○	○	○

Zuversicht und Zufriedenheit

34 Wie zuversichtlich bist Du hinsichtlich folgender Bereiche deines Lebens?
Bitte wählen Sie die zutreffende Antwort für jeden Punkt aus:

	gar nicht zuversichtlich	ein wenig zuversichtlich	zuversichtlich	sehr zuversichtlich
Schulischer Erfolg	○	○	○	○
Berufliche Zukunft	○	○	○	○
Persönliches Wohlergehen	○	○	○	○
Finanzielles	○	○	○	○

Verhältnisse

35 Wie zufrieden bist Du zum jetzigen Zeitpunkt mit deiner Lebenssituation?
Bitte wählen Sie nur eine der folgenden Antworten aus:

- ◯ sehr unzufrieden
- ◯ eher unzufrieden
- ◯ zufrieden
- ◯ sehr zufrieden

36 Wie wichtig sind Dir Deine Leistungen in der Schule?
Bitte wählen Sie nur eine der folgenden Antworten aus:

- ◯ überhaupt nicht wichtig
- ◯ nicht wichtig
- ◯ wichtig
- ◯ sehr wichtig

37 Wie wichtig ist Dir die Schule allgemein?
Bitte wählen Sie nur eine der folgenden Antworten aus:

- ◯ überhaupt nicht wichtig
- ◯ nicht wichtig
- ◯ wichtig
- ◯ sehr wichtig

38 Wie wichtig ist Dir deine Lehre?
Beantworten Sie diese Frage nur, wenn folgende Bedingungen erfüllt sind:
Antwort war 'Berufsfachschule/Lehre' bei Frage 2 (Welche Schule besuchst Du?)
Bitte wählen Sie nur eine der folgenden Antworten aus:

- ◯ überhaupt nicht wichtig
- ◯ nicht wichtig
- ◯ wichtig
- ◯ sehr wichtig

Konsum Medikamente

39 Hast Du schon einmal in deinem Leben eines oder mehrere der unten aufgeführten Medikamente/Substanzen eingenommen, *ohne dies aufgrund einer bestimmten Erkrankung* zu tun und wenn ja als *Freizeitkonsum*[1] und/oder zur *Leistungssteigerung*[2]?

[1] Freizeitkonsum betrifft den Konsum im Ausgang, an Parties, um länger tanzen/wach bleiben zu können, als Genussmittel oder zur Entspannung.
[2] Leistungssteigerung betrifft den Konsum mit der Absicht in der Schule oder im Beruf besser zu sein. Zum Beispiel, um in der Prüfungszeit bessere Leistungen zu erbringen, mit dem Schul- oder Arbeitsstress klarzukommen, um besser und länger lernen oder arbeiten zu können, oder um vor der Prüfung oder vor einem Stresstag besser schlafen und entspannen zu können.

Bitte wählen Sie die zutreffende Antwort für jeden Punkt aus:

	nein	nur Freizeit-	nur zur Leistungs-	Freizeitkonsum[1]

		konsum[1]	steigerung[2]	UND Leistungs-steigerung[2]
**Methylphenidat** (zum Beispiel Ritalin®, Concerta®, Medikinet®, Focalin®, Strattera®)	○	○	○	○
**Modafinil** (zum Beispiel Modasomil®)	○	○	○	○
**Hustenmittel**	○	○	○	○
**Aktivierende Antidepressiva** (zum Beispiel Edronax®, Wellbutrin®, Efexor®, Cymbalta®, Fluctine®/Fluoxetin®, Seropram®/Citalopram®, Cipralex®, Zoloft®/Sertralin®)	○	○	○	○
**Antidementiva** (zum Beispiel Aricept®, Axura®, Reminyl®, Exelon®)	○	○	○	○
**Beruhigungs- und Schlafmittel, Hypnotica** (zum Beispiel Remeron®, Sonata®, Stilnox®, Imovane®, Dalmadorm®, Noctamid®, Ergocalm Tabs®, Remestan®, Halcion®, Dolestan®, Chloraldurat 500®, Temesta®, Valium®, Xanax®)	○	○	○	○
**Pflanzliche Beruhigungsmittel** (zum Beispiel Baldriparan®, Faros®, Jasin®, Remotiv®, Zeller Entspannung®, Zeller Herz-Nerven®, Johanniskraut)	○	○	○	○
**Betablocker** (zum Beispiel Beloc ZOK®, MetoZerok®, Inderal®)	○	○	○	○

**Stärkungsmittel, Nahrungsergänzung und Vitaminpräparate** (zum Beispiel Berocca®, Dynamiasan®, Gincosan®, Strath®, Supradyn®, Tonoglutal®, Omega 3-Fettsäure-Präparate)	○	○	○	○

Methylphenidat in der Freizeit

Diese Fragen betreffen deinen Konsum von **Methylphenidat** (zum Beispiel Ritalin®, Concerta®, Medikinet®, Focalin®, Strattera®) in der **Freizeit**

40 Wie häufig hast Du diese Substanz in den letzten 12 Monaten in der Freizeit z.B. im Ausgang, an Parties etc. durchschnittlich verwendet?
Bitte wählen Sie nur eine der folgenden Antworten aus:

- ○ Täglich
- ○ Wöchentlich
- ○ Alle 14 Tage
- ○ Monatlich
- ○ 1-2 Mal
- ○ Nie

41 Wie häufig hast Du diese Substanz in den letzten 30 Tagen in der Freizeit z.B. im Ausgang, an Parties etc. durchschnittlich verwendet?
Bitte wählen Sie nur eine der folgenden Antworten aus:

- ○ An 20 oder mehr Tagen
- ○ An 10-19 Tagen
- ○ An 4-9 Tagen
- ○ An 1-3 Tagen
- ○ Gar nicht

Methylphenidat zur Verbesserung deiner Gehirnleistung

Diese Fragen betreffen deinen Konsum von **Methylphenidat** (zum Beispiel Ritalin®, Concerta®, Medikinet®, Focalin®, Strattera®) zur **Verbesserung deiner Gehirnleistung**. Also um bessere Leistungen zu erbringen, mit dem Schul- oder Arbeitsstress klarzukommen, um besser und länger lernen oder arbeiten zu können oder um vor der Prüfung oder einem Stresstag besser schlafen und entspannen zu können?

42 Wie häufig hast Du diese Substanz in den letzten 12 Monaten durchschnittlich verwendet, um deine Gehirnleistung für die Berufsvorbereitungsschule/Berufsfachschule/Kantonsschule zu verbessern?
Bitte wählen Sie nur eine der folgenden Antworten aus:

- ○ Täglich
- ○ Wöchentlich
- ○ Alle 14 Tage

○ Monatlich
○ 1-2 Mal
○ Nie

43 Wie häufig hast Du diese Substanz in den letzten 30 Tagen (ohne Urlaub) verwendet, um deine Gehirnleistung für die Berufsvorbereitungsschule/Berufsfachschule/Kantonsschule zu verbessern?
Bitte wählen Sie nur eine der folgenden Antworten aus:

○ An 20 oder mehr Tagen
○ An 10-19 Tagen
○ An 4-9 Tagen
○ An 1-3 Tagen
○ Gar nicht

44 Was war der Grund für die Einnahme?
Bitte wählen Sie alle zutreffenden Antworten aus:

☐ Wach bleiben/länger lernen
☐ Sich konzentrieren/Aufmerksam sein
☐ Gedächtnis verbessern
☐ High sein (Rausch erzeugen)
☐ Gefühl geniessen
☐ Mit dem Druck klar kommen
☐ Besser schlafen können
☐ Allgemeine Entspannung
☐ Noten verbessern
☐ Prüfungsangst während der Prüfung bekämpfen
☐ Weil andere es tun
☐ Sonstiges:

45 Wurden Deine Erwartungen an die Wirkung der Substanz erfüllt?
Bitte wählen Sie nur eine der folgenden Antworten aus:

○ Ja
○ Nein
○ Teilweise
○ Ich weiss nicht

46 Würdest Du es in einer ähnlichen Situation wieder einnehmen?
Bitte wählen Sie nur eine der folgenden Antworten aus:

○ Ja
○ Nein
○ Vielleicht
○ Ich weiss nicht

Modafinil in der Freizeit

Diese Fragen betreffen deinen Konsum von **Modafinil** (zum Beispiel Modasomil®) in der **Freizeit**

47 Wie häufig hast Du diese Substanz in den letzten 12 Monaten in der Freizeit z.B. im Ausgang, an Parties etc. durchschnittlich verwendet?
Bitte wählen Sie nur eine der folgenden Antworten aus:

- ○ Täglich
- ○ Wöchentlich
- ○ Alle 14 Tage
- ○ Monatlich
- ○ 1-2 Mal
- ○ Nie

48 Wie häufig hast Du diese Substanz in den letzten 30 Tagen in der Freizeit z.B. im Ausgang, an Parties etc. durchschnittlich verwendet?
Bitte wählen Sie nur eine der folgenden Antworten aus:

- ○ An 20 oder mehr Tagen
- ○ An 10-19 Tagen
- ○ An 4-9 Tagen
- ○ An 1-3 Tagen
- ○ Gar nicht

Modafinil zur Verbesserung deiner Gehirnleistung

Diese Fragen betreffen deinen Konsum von **Modafinil** (zum Beispiel Modasomil®) zur **Verbesserung deiner Gehirnleistung**. Also um bessere Leistungen zu erbringen, mit dem Schul- oder Arbeitsstress klarzukommen, um besser und länger lernen oder arbeiten zu können oder um vor der Prüfung oder einem Stresstag besser schlafen und entspannen zu können?

49 Wie häufig hast Du diese Substanz in den letzten 12 Monaten durchschnittlich verwendet, um deine Gehirnleistung für die Berufsvorbereitungsschule/Berufsfachschule/Kantonsschule zu verbessern?
Bitte wählen Sie nur eine der folgenden Antworten aus:

- ○ Täglich
- ○ Wöchentlich
- ○ Alle 14 Tage
- ○ Monatlich
- ○ 1-2 Mal
- ○ Nie

50 Wie häufig hast Du diese Substanz in den letzten 30 Tagen (ohne Urlaub) verwendet, um deine Gehirnleistung für die Berufsvorbereitungsschule/Berufsfachschule/Kantonsschule zu verbessern?
Bitte wählen Sie nur eine der folgenden Antworten aus:

- ○ An 20 oder mehr Tagen
- ○ An 10-19 Tagen
- ○ An 4-9 Tagen

○ An 1-3 Tagen

○ Gar nicht

51 Was war der Grund für die Einnahme?
Bitte wählen Sie alle zutreffenden Antworten aus:

☐ Wach bleiben/länger lernen

☐ Sich konzentrieren/Aufmerksam sein

☐ Gedächtnis verbessern

☐ High sein (Rausch erzeugen)

☐ Gefühl geniessen

☐ Mit dem Druck klar kommen

☐ Besser schlafen können

☐ Allgemeine Entspannung

☐ Noten verbessern

☐ Prüfungsangst während der Prüfung bekämpfen

☐ Weil andere es tun

☐ Sonstiges:

52 Wurden Deine Erwartungen an die Wirkung der Substanz erfüllt?
Bitte wählen Sie nur eine der folgenden Antworten aus:

○ Ja

○ Nein

○ Teilweise

○ Ich weiss nicht

53 Würdest Du es in einer ähnlichen Situation wieder einnehmen?
Bitte wählen Sie nur eine der folgenden Antworten aus:

○ Ja

○ Nein

○ Vielleicht

○ Ich weiss nicht

Hustenmittel in der Freizeit

Diese Fragen betreffen deinen Konsum von **Hustenmittel** in der **Freizeit**.

54 Wie häufig hast Du diese Substanz in den letzten 12 Monaten in der Freizeit z.B. im Ausgang, an Parties etc. durchschnittlich verwendet?

Bitte wählen Sie nur eine der folgenden Antworten aus:

○ Täglich

○ Wöchentlich

○ Alle 14 Tage

○ Monatlich

◯ 1-2 Mal
◯ Nie

55 Wie häufig hast Du diese Substanz in den letzten 30 Tagen in der Freizeit z.B. im Ausgang, an Parties etc. durchschnittlich verwendet?
Bitte wählen Sie nur eine der folgenden Antworten aus:
◯ An 20 oder mehr Tagen
◯ An 10-19 Tagen
◯ An 4-9 Tagen
◯ An 1-3 Tagen
◯ Gar nicht

Hustenmittel zur Verbesserung deiner Gehirnleistung

Diese Fragen betreffen deinen Konsum von **Hustenmittel** zur **Verbesserung deiner Gehirnleistung**. Also um bessere Leistungen zu erbringen, mit dem Schul- oder Arbeitsstress klarzukommen, um besser und länger lernen oder arbeiten zu können oder um vor der Prüfung oder einem Stresstag besser schlafen und entspannen zu können?

56 Wie häufig hast Du diese Substanz in den letzten 12 Monaten durchschnittlich verwendet, um deine Gehirnleistung für die Berufsvorbereitungsschule/Berufsfachschule/Kantonsschule zu verbessern?
Bitte wählen Sie nur eine der folgenden Antworten aus:
◯ Täglich
◯ Wöchentlich
◯ Alle 14 Tage
◯ Monatlich
◯ 1-2 Mal
◯ Nie

57 Wie häufig hast Du diese Substanz in den letzten 30 Tagen (ohne Urlaub) verwendet, um deine Gehirnleistung für die Berufsvorbereitungsschule/Berufsfachschule/Kantonsschule zu verbessern?
Bitte wählen Sie nur eine der folgenden Antworten aus:
◯ An 20 oder mehr Tagen
◯ An 10-19 Tagen
◯ An 4-9 Tagen
◯ An 1-3 Tagen
◯ Gar nicht

58 Was war der Grund für die Einnahme?
Bitte wählen Sie alle zutreffenden Antworten aus:
☐ Wach bleiben/länger lernen
☐ Sich konzentrieren/Aufmerksam sein
☐ Gedächtnis verbessern
☐ High sein (Rausch erzeugen)

- [ ] Gefühl geniessen
- [ ] Mit dem Druck klar kommen
- [ ] Besser schlafen können
- [ ] Allgemeine Entspannung
- [ ] Noten verbessern
- [ ] Prüfungsangst während der Prüfung bekämpfen
- [ ] Weil andere es tun
- [ ] Sonstiges:

59 Wurden Deine Erwartungen an die Wirkung der Substanz erfüllt?
Bitte wählen Sie nur eine der folgenden Antworten aus:

- ○ Ja
- ○ Nein
- ○ Teilweise
- ○ Ich weiss nicht

60 Würdest Du es in einer ähnlichen Situation wieder einnehmen?
Bitte wählen Sie nur eine der folgenden Antworten aus:

- ○ Ja
- ○ Nein
- ○ Vielleicht
- ○ Ich weiss nicht

Aktivierende Antidepressiva in der Freizeit

Diese Fragen betreffen deinen Konsum von **aktivierenden Antidepressiva** (zum Beispiel Edronax®, Wellbutrin®, Efexor®, Cymbalta®, Fluctine®/Fluoxetin®, Seropram®/Citalopram®, Cipralex®, Zoloft®/Sertralin®) in der **Freizeit**

61 Wie häufig hast Du diese Substanz in den letzten 12 Monaten in der Freizeit z.B. im Ausgang, an Parties etc. durchschnittlich verwendet?
Bitte wählen Sie nur eine der folgenden Antworten aus:

- ○ Täglich
- ○ Wöchentlich
- ○ Alle 14 Tage
- ○ Monatlich
- ○ 1-2 Mal
- ○ Nie

62 Wie häufig hast Du diese Substanz in den letzten 30 Tagen in der Freizeit z.B. im Ausgang, an Parties etc. durchschnittlich verwendet?
Bitte wählen Sie nur eine der folgenden Antworten aus:

- ○ An 20 oder mehr Tagen
- ○ An 10-19 Tagen

○ An 4-9 Tagen
○ An 1-3 Tagen
○ Gar nicht

Aktivierende Antidepressiva zur Verbesserung deiner Gehirnleistung

Diese Fragen betreffen deinen Konsum von **aktivierenden Antidepressiva** (zum Beispiel Edronax®, Wellbutrin®, Efexor®, Cymbalta®, Fluctine®/Fluoxetin®, Seropram®/Citalopram®, Cipralex®, Zoloft®/Sertralin®) zur **Verbesserung deiner Gehirnleistung**. Also um bessere Leistungen zu erbringen, mit dem Schul- oder Arbeitsstress klarzukommen, um besser und länger lernen oder arbeiten zu können oder um vor der Prüfung oder einem Stresstag besser schlafen und entspannen zu können?

63 Wie häufig hast Du diese Substanz in den letzten 12 Monaten durchschnittlich verwendet, um deine Gehirnleistung für die Berufsvorbereitungsschule/Berufsfachschule/Kantonsschule zu verbessern?
Bitte wählen Sie nur eine der folgenden Antworten aus:
○ Täglich
○ Wöchentlich
○ Alle 14 Tage
○ Monatlich
○ 1-2 Mal
○ Nie

64 Wie häufig hast Du diese Substanz in den letzten 30 Tagen (ohne Urlaub) verwendet, um deine Gehirnleistung für die Berufsvorbereitungsschule/Berufsfachschule/Kantonsschule zu verbessern?
Bitte wählen Sie nur eine der folgenden Antworten aus:
○ An 20 oder mehr Tagen
○ An 10-19 Tagen
○ An 4-9 Tagen
○ An 1-3 Tagen
○ Gar nicht

65 Was war der Grund für die Einnahme?
Bitte wählen Sie alle zutreffenden Antworten aus:
☐ Wach bleiben/länger lernen
☐ Sich konzentrieren/Aufmerksam sein
☐ Gedächtnis verbessern
☐ High sein (Rausch erzeugen)
☐ Gefühl geniessen
☐ Mit dem Druck klar kommen
☐ Besser schlafen können
☐ Allgemeine Entspannung
☐ Noten verbessern
☐ Prüfungsangst während der Prüfung bekämpfen
☐ Weil andere es tun

☐ Sonstiges:

66 Wurden Deine Erwartungen an die Wirkung der Substanz erfüllt?
Bitte wählen Sie nur eine der folgenden Antworten aus:

- ○ Ja
- ○ Nein
- ○ Teilweise
- ○ Ich weiss nicht

67 Würdest Du es in einer ähnlichen Situation wieder einnehmen?
Bitte wählen Sie nur eine der folgenden Antworten aus:

- ○ Ja
- ○ Nein
- ○ Vielleicht
- ○ Ich weiss nicht

Antidementiva in der Freizeit

Diese Fragen betreffen deinen Konsum von **Antidementiva** (zum Beispiel Aricept®, Axura®, Reminyl®, Exelon®) in der **Freizeit**

68 Wie häufig hast Du diese Substanz in den letzten 12 Monaten in der Freizeit z.B. im Ausgang, an Parties etc. durchschnittlich verwendet?
Bitte wählen Sie nur eine der folgenden Antworten aus:

- ○ Täglich
- ○ Wöchentlich
- ○ Alle 14 Tage
- ○ Monatlich
- ○ 1-2 Mal
- ○ Nie

69 Wie häufig hast Du diese Substanz in den letzten 30 Tagen in der Freizeit z.B. im Ausgang, an Parties etc. durchschnittlich verwendet?
Bitte wählen Sie nur eine der folgenden Antworten aus:

- ○ An 20 oder mehr Tagen
- ○ An 10-19 Tagen
- ○ An 4-9 Tagen
- ○ An 1-3 Tagen
- ○ Gar nicht

Antidementiva zur Verbesserung deiner Gehirnleistung

Diese Fragen betreffen deinen Konsum von **Antidementiva** (zum Beispiel Aricept®, Axura®, Reminyl®, Exelon®) zur **Verbesserung deiner Gehirnleistung**. Also um bessere Leistungen zu erbringen, mit dem Schul- oder Arbeitsstress klarzukommen, um besser und länger lernen oder arbeiten zu können oder um vor der Prüfung

oder einem Stresstag besser schlafen und entspannen zu können?

70 Wie häufig hast Du diese Substanz in den letzten 12 Monaten durchschnittlich verwendet, um deine Gehirnleistung für die Berufsvorbereitungsschule/Berufsfachschule/Kantonsschule zu verbessern?
Bitte wählen Sie nur eine der folgenden Antworten aus:

- ○ Täglich
- ○ Wöchentlich
- ○ Alle 14 Tage
- ○ Monatlich
- ○ 1-2 Mal
- ○ Nie

71 Wie häufig hast Du diese Substanz in den letzten 30 Tagen (ohne Urlaub) verwendet, um deine Gehirnleistung für die Berufsvorbereitungsschule/Berufsfachschule/Kantonsschule zu verbessern?
Bitte wählen Sie nur eine der folgenden Antworten aus:

- ○ An 20 oder mehr Tagen
- ○ An 10-19 Tagen
- ○ An 4-9 Tagen
- ○ An 1-3 Tagen
- ○ Gar nicht

72 Was war der Grund für die Einnahme?
Bitte wählen Sie alle zutreffenden Antworten aus:

- ☐ Wach bleiben/länger lernen
- ☐ Sich konzentrieren/Aufmerksam sein
- ☐ Gedächtnis verbessern
- ☐ High sein (Rausch erzeugen)
- ☐ Gefühl geniessen
- ☐ Mit dem Druck klar kommen
- ☐ Besser schlafen können
- ☐ Allgemeine Entspannung
- ☐ Noten verbessern
- ☐ Prüfungsangst während der Prüfung bekämpfen
- ☐ Weil andere es tun
- ☐ Sonstiges:

73 Wurden Deine Erwartungen an die Wirkung der Substanz erfüllt?
Bitte wählen Sie nur eine der folgenden Antworten aus:

- ○ Ja
- ○ Nein
- ○ Teilweise
- ○ Ich weiss nicht

74 Würdest Du es in einer ähnlichen Situation wieder einnehmen?
Bitte wählen Sie nur eine der folgenden Antworten aus:

- Ja
- Nein
- Vielleicht
- Ich weiss nicht

Beruhigungs- und Schlafmittel, Hypnotica in der Freizeit

Diese Fragen betreffen deinen Konsum von **Beruhigungs- und Schlafmittel, Hypnotica** (zum Beispiel Remeron®, Sonata®, Stilnox®, Imovane®, Dalmadorm®, Noctamid®, Ergocalm Tabs®, Remestan®, Halcion®, Dolestan®, Chloraldurat 500®, Temesta®, Valium®, Xanax®) in der **Freizeit**

75 Wie häufig hast Du diese Substanz in den letzten 12 Monaten in der Freizeit z.B. im Ausgang, an Parties etc. durchschnittlich verwendet?
Bitte wählen Sie nur eine der folgenden Antworten aus:

- Täglich
- Wöchentlich
- Alle 14 Tage
- Monatlich
- 1-2 Mal
- Nie

76 Wie häufig hast Du diese Substanz in den letzten 30 Tagen in der Freizeit z.B. im Ausgang, an Parties etc. durchschnittlich verwendet?
Bitte wählen Sie nur eine der folgenden Antworten aus:

- An 20 oder mehr Tagen
- An 10-19 Tagen
- An 4-9 Tagen
- An 1-3 Tagen
- Gar nicht

Beruhigungs- und Schlafmittel, Hypnotica zur Verbesserung deiner Gehirnleistung

Diese Fragen betreffen deinen Konsum von **Beruhigungs- und Schlafmittel, Hypnotica** (zum Beispiel Remeron®, Sonata®, Stilnox®, Imovane®, Dalmadorm®, Noctamid®, Ergocalm Tabs®, Remestan®, Halcion®, Dolestan®, Chloraldurat 500®, Temesta®, Valium®, Xanax®) zur **Verbesserung deiner Gehirnleistung**. Also um bessere Leistungen zu erbringen, mit dem Schul- oder Arbeitsstress klarzukommen, um besser und länger lernen oder arbeiten zu können oder um vor der Prüfung oder einem Stresstag besser schlafen und entspannen zu können?

77 Wie häufig hast Du diese Substanz in den letzten 12 Monaten durchschnittlich verwendet, um deine Gehirnleistung für die Berufsvorbereitungsschule/Berufsfachschule/Kantonsschule zu verbessern?
Bitte wählen Sie nur eine der folgenden Antworten aus:

- Täglich
- Wöchentlich

○ Alle 14 Tage
○ Monatlich
○ 1-2 Mal
○ Nie

78 Wie häufig hast Du diese Substanz in den letzten 30 Tagen (ohne Urlaub) verwendet, um deine Gehirnleistung für die Berufsvorbereitungsschule/Berufsfachschule/Kantonsschule zu verbessern?
Bitte wählen Sie nur eine der folgenden Antworten aus:

○ An 20 oder mehr Tagen
○ An 10-19 Tagen
○ An 4-9 Tagen
○ An 1-3 Tagen
○ Gar nicht

79 Was war der Grund für die Einnahme?
Bitte wählen Sie alle zutreffenden Antworten aus:

☐ Wach bleiben/länger lernen
☐ Sich konzentrieren/Aufmerksam sein
☐ Gedächtnis verbessern
☐ High sein (Rausch erzeugen)
☐ Gefühl geniessen
☐ Mit dem Druck klar kommen
☐ Besser schlafen können
☐ Allgemeine Entspannung
☐ Noten verbessern
☐ Prüfungsangst während der Prüfung bekämpfen
☐ Weil andere es tun
☐ Sonstiges:

80 Wurden Deine Erwartungen an die Wirkung der Substanz erfüllt?
Bitte wählen Sie nur eine der folgenden Antworten aus:

○ Ja
○ Nein
○ Teilweise
○ Ich weiss nicht

81 Würdest Du es in einer ähnlichen Situation wieder einnehmen?
Bitte wählen Sie nur eine der folgenden Antworten aus:

○ Ja
○ Nein
○ Vielleicht
○ Ich weiss nicht

Pflanzliche Beruhigungsmittel in der Freizeit

Diese Fragen betreffen deinen Konsum von **Pflanzliche Beruhigungsmittel** (zum Beispiel Baldriparan®, Faros®, Jasin®, Remotiv®, Zeller Entspannung®, Zeller Herz-Nerven®, Johanniskraut) in der **Freizeit**

82 Wie häufig hast Du diese Substanz in den letzten 12 Monaten in der Freizeit z.B. im Ausgang, an Parties etc. durchschnittlich verwendet?
Bitte wählen Sie nur eine der folgenden Antworten aus:

- ◯ Täglich
- ◯ Wöchentlich
- ◯ Alle 14 Tage
- ◯ Monatlich
- ◯ 1-2 Mal
- ◯ Nie

83 Wie häufig hast Du diese Substanz in den letzten 30 Tagen in der Freizeit z.B. im Ausgang, an Parties etc. durchschnittlich verwendet?
Bitte wählen Sie nur eine der folgenden Antworten aus:

- ◯ An 20 oder mehr Tagen
- ◯ An 10-19 Tagen
- ◯ An 4-9 Tagen
- ◯ An 1-3 Tagen
- ◯ Gar nicht

Pflanzliche Beruhigungsmittel zur Verbesserung deiner Gehirnleistung

Diese Fragen betreffen deinen Konsum von **Pflanzliche Beruhigungsmittel** (zum Beispiel Baldriparan®, Faros®, Jasin®, Remotiv®, Zeller Entspannung®, Zeller Herz-Nerven®, Johanniskraut) zur **Verbesserung deiner Gehirnleistung**. Also um bessere Leistungen zu erbringen, mit dem Schul- oder Arbeitsstress klarzukommen, um besser und länger lernen oder arbeiten zu können oder um vor der Prüfung oder einem Stresstag besser schlafen und entspannen zu können?

84 Wie häufig hast Du diese Substanz in den letzten 12 Monaten durchschnittlich verwendet, um deine Gehirnleistung für die Berufsvorbereitungsschule/Berufsfachschule/Kantonsschule zu verbessern?
Bitte wählen Sie nur eine der folgenden Antworten aus:

- ◯ Täglich
- ◯ Wöchentlich
- ◯ Alle 14 Tage
- ◯ Monatlich
- ◯ 1-2 Mal
- ◯ Nie

85 Wie häufig hast Du diese Substanz in den letzten 30 Tagen (ohne Urlaub) verwendet, um deine Gehirnleistung für die Berufsvorbereitungsschule/Berufsfachschule/Kantonsschule zu verbessern?
Bitte wählen Sie nur eine der folgenden Antworten aus:

- ◯ An 20 oder mehr Tagen

- ○ An 10-19 Tagen
- ○ An 4-9 Tagen
- ○ An 1-3 Tagen
- ○ Gar nicht

86 Was war der Grund für die Einnahme?
Bitte wählen Sie alle zutreffenden Antworten aus:

- ☐ Wach bleiben/länger lernen
- ☐ Sich konzentrieren/Aufmerksam sein
- ☐ Gedächtnis verbessern
- ☐ High sein (Rausch erzeugen)
- ☐ Gefühl geniessen
- ☐ Mit dem Druck klar kommen
- ☐ Besser schlafen können
- ☐ Allgemeine Entspannung
- ☐ Noten verbessern
- ☐ Prüfungsangst während der Prüfung bekämpfen
- ☐ Weil andere es tun
- ☐ Sonstiges:

87 Wurden Deine Erwartungen an die Wirkung der Substanz erfüllt?
Bitte wählen Sie nur eine der folgenden Antworten aus:

- ○ Ja
- ○ Nein
- ○ Teilweise
- ○ Ich weiss nicht

88 Würdest Du es in einer ähnlichen Situation wieder einnehmen?
Bitte wählen Sie nur eine der folgenden Antworten aus:

- ○ Ja
- ○ Nein
- ○ Vielleicht
- ○ Ich weiss nicht

Betablocker in der Freizeit

Diese Fragen betreffen deinen Konsum von **Betablocker** (zum Beispiel Beloc ZOK®, MetoZerok®, Inderal®) in der **Freizeit**

89 Wie häufig hast Du diese Substanz in den letzten 12 Monaten in der Freizeit z.B. im Ausgang, an Parties etc. durchschnittlich verwendet?
Bitte wählen Sie nur eine der folgenden Antworten aus:

- ○ Täglich

- ○ Wöchentlich
- ○ Alle 14 Tage
- ○ Monatlich
- ○ 1-2 Mal
- ○ Nie

90 Wie häufig hast Du diese Substanz in den letzten 30 Tagen in der Freizeit z.B. im Ausgang, an Parties etc. durchschnittlich verwendet?
Bitte wählen Sie nur eine der folgenden Antworten aus:

- ○ An 20 oder mehr Tagen
- ○ An 10-19 Tagen
- ○ An 4-9 Tagen
- ○ An 1-3 Tagen
- ○ Gar nicht

Betablocker zur Verbesserung deiner Gehirnleistung

Diese Fragen betreffen deinen Konsum von **Betablocker** (zum Beispiel Beloc ZOK®, MetoZerok®, Inderal®) zur **Verbesserung deiner Gehirnleistung**. Also um bessere Leistungen zu erbringen, mit dem Schul- oder Arbeitsstress klarzukommen, um besser und länger lernen oder arbeiten zu können oder um vor der Prüfung oder einem Stresstag besser schlafen und entspannen zu können?

91 Wie häufig hast Du diese Substanz in den letzten 12 Monaten durchschnittlich verwendet, um deine Gehirnleistung für die Berufsvorbereitungsschule/Berufsfachschule/Kantonsschule zu verbessern?
Bitte wählen Sie nur eine der folgenden Antworten aus:

- ○ Täglich
- ○ Wöchentlich
- ○ Alle 14 Tage
- ○ Monatlich
- ○ 1-2 Mal
- ○ Nie

92 Wie häufig hast Du diese Substanz in den letzten 30 Tagen (ohne Urlaub) verwendet, um deine Gehirnleistung für die Berufsvorbereitungsschule/Berufsfachschule/Kantonsschule zu verbessern?
Bitte wählen Sie nur eine der folgenden Antworten aus:

- ○ An 20 oder mehr Tagen
- ○ An 10-19 Tagen
- ○ An 4-9 Tagen
- ○ An 1-3 Tagen
- ○ Gar nicht

93 Was war der Grund für die Einnahme?
Bitte wählen Sie alle zutreffenden Antworten aus:

- ☐ Wach bleiben/länger lernen

☐ Sich konzentrieren/Aufmerksam sein
☐ Gedächtnis verbessern
☐ High sein (Rausch erzeugen)
☐ Gefühl geniessen
☐ Mit dem Druck klar kommen
☐ Besser schlafen können
☐ Allgemeine Entspannung
☐ Noten verbessern
☐ Prüfungsangst während der Prüfung bekämpfen
☐ Weil andere es tun
☐ Sonstiges:

94 Wurden Deine Erwartungen an die Wirkung der Substanz erfüllt?
Bitte wählen Sie nur eine der folgenden Antworten aus:
○ Ja
○ Nein
○ Teilweise
○ Ich weiss nicht

95 Würdest Du es in einer ähnlichen Situation wieder einnehmen?
Bitte wählen Sie nur eine der folgenden Antworten aus:
○ Ja
○ Nein
○ Vielleicht
○ Ich weiss nicht

Stärkungsmittel, Nahrungsergänzung und Vitaminpräparate in der Freizeit

Diese Fragen betreffen deinen Konsum von **Stärkungsmittel, Nahrungsergänzung und Vitaminpräparate** (zum Beispiel Berocca®, Dynamiasan®, Gincosan®, Strath®, Supradyn®, Tonoglutal®, Omega 3-Fettsäure-Präparate) in der **Freizeit**

96 Wie häufig hast Du diese Substanz in den letzten 12 Monaten in der Freizeit z.B. im Ausgang, an Parties etc. durchschnittlich verwendet?
Bitte wählen Sie nur eine der folgenden Antworten aus:
○ Täglich
○ Wöchentlich
○ Alle 14 Tage
○ Monatlich
○ 1-2 Mal
○ Nie

97 Wie häufig hast Du diese Substanz in den letzten 30 Tagen in der Freizeit z.B. im Ausgang, an Parties

etc. durchschnittlich verwendet?
Bitte wählen Sie nur eine der folgenden Antworten aus:

- An 20 oder mehr Tagen
- An 10-19 Tagen
- An 4-9 Tagen
- An 1-3 Tagen
- Gar nicht

Stärkungsmittel, Nahrungsergänzung und Vitaminpräparate zur Verbesserung deiner Gehirnleistung

Diese Fragen betreffen deinen Konsum von **Stärkungsmittel, Nahrungsergänzung und Vitaminpräparaten** (zum Beispiel Berocca®, Dynamiasan®, Gincosan®, Strath®, Supradyn®, Tonoglutal®, Omega 3-Fettsäure-Präparate) zur **Verbesserung deiner Gehirnleistung**. Also um bessere Leistungen zu erbringen, mit dem Schul- oder Arbeitsstress klarzukommen, um besser und länger lernen oder arbeiten zu können oder um vor der Prüfung oder einem Stresstag besser schlafen und entspannen zu können?

98 Wie häufig hast Du diese Substanz in den letzten 12 Monaten durchschnittlich verwendet, um deine Gehirnleistung für die Berufsvorbereitungsschule/Berufsfachschule/Kantonsschule zu verbessern?
Bitte wählen Sie nur eine der folgenden Antworten aus:

- Täglich
- Wöchentlich
- Alle 14 Tage
- Monatlich
- 1-2 Mal
- Nie

99 Wie häufig hast Du diese Substanz in den letzten 30 Tagen (ohne Urlaub) verwendet, um deine Gehirnleistung für die Berufsvorbereitungsschule/Berufsfachschule/Kantonsschule zu verbessern?
Bitte wählen Sie nur eine der folgenden Antworten aus:

- An 20 oder mehr Tagen
- An 10-19 Tagen
- An 4-9 Tagen
- An 1-3 Tagen
- Gar nicht

100 Was war der Grund für die Einnahme?
Bitte wählen Sie alle zutreffenden Antworten aus:

- [ ] Wach bleiben/länger lernen
- [ ] Sich konzentrieren/Aufmerksam sein
- [ ] Gedächtnis verbessern
- [ ] High sein (Rausch erzeugen)
- [ ] Gefühl geniessen
- [ ] Mit dem Druck klar kommen

☐ Besser schlafen können

☐ Allgemeine Entspannung

☐ Noten verbessern

☐ Prüfungsangst während der Prüfung bekämpfen

☐ Weil andere es tun

☐ Sonstiges:

101 Wurden Deine Erwartungen an die Wirkung der Substanz erfüllt?
Bitte wählen Sie nur eine der folgenden Antworten aus:

○ Ja

○ Nein

○ Teilweise

○ Ich weiss nicht

102 Würdest Du es in einer ähnlichen Situation wieder einnehmen?
Bitte wählen Sie nur eine der folgenden Antworten aus:

○ Ja

○ Nein

○ Vielleicht

○ Ich weiss nicht

Konsum psychoaktive Substanzen

103 Hast Du schon einmal in deinem Leben eines oder mehrere der unten aufgeführten psychoaktiven Substanzen eingenommen, und wenn ja als *Freizeitkonsum*[1] und/oder zur *Leistungssteigerung*[2]?

[1] Freizeitkonsum betrifft den Konsum im Ausgang, an Parties, um länger tanzen/wach bleiben zu können, als Genussmittel oder zur Entspannung.
[2] Leistungssteigerung betrifft den Konsum mit der Absicht in der Schule oder im Beruf besser zu sein. Zum Beispiel, um in der Prüfungszeit bessere Leistungen zu erbringen, mit dem Schul- oder Arbeitsstress klarzukommen, um besser und länger lernen oder arbeiten zu können, oder um vor der Prüfung oder vor einem Stresstag besser schlafen und entspannen zu können.

Bitte wählen Sie die zutreffende Antwort für jeden Punkt aus:

	nein	nur Freizeit-konsum[1]	nur zur Leistungs-steigerung[2]	Freizeitkonsum[1] UND Leistungs-steigerung[2]
**Alkohol**	○	○	○	○
**Tabak** (zum Beispiel Zigaretten, Zigarren, Pfeife, Schnupftabak, Kautabak)	○	○	○	○
**Hanfprodukte** (zum Beispiel Cannabis	○	○	○	○

(Marihuana), Hasch, Haschöl)				
**Kokain**	○	○	○	○
**Amphetamine** (Speed)	○	○	○	○
**Ecstasy** (MDMA)	○	○	○	○
**Kaffee**	○	○	○	○
**Koffeintabletten** (zum Beispiel Coffeinum N®, Percoffedrinol®)	○	○	○	○
**Energy Drinks** (zum Beispiel Red Bull®, guaranahaltige Getränke)	○	○	○	○
**Andere**	○	○	○	○

Alkohol in der Freizeit

Diese Fragen betreffen deinen Konsum von **Alkohol** in der **Freizeit**, zum Beispiel im Ausgang oder auf Partys.

104 Wie häufig hast Du diese Substanz in den letzten 12 Monaten in der Freizeit z.B. im Ausgang, an Parties etc. durchschnittlich verwendet?
Bitte wählen Sie nur eine der folgenden Antworten aus:

○ Täglich
○ Wöchentlich
○ Alle 14 Tage
○ Monatlich
○ 1-2 Mal
○ Nie

105 Wie häufig hast Du diese Substanz in den letzten 30 Tagen in der Freizeit z.B. im Ausgang, an Parties etc. durchschnittlich verwendet?
Bitte wählen Sie nur eine der folgenden Antworten aus:

○ An 20 oder mehr Tagen
○ An 10-19 Tagen
○ An 4-9 Tagen
○ An 1-3 Tagen
○ Gar nicht

106 Wie viele Standarddrinks[1] pro Tag bei dem Du Alkohol getrunken hast, hast du durchschnittlich im letzten Monat getrunken?

Bitte wählen Sie nur eine der folgenden Antworten aus:

- 0 oder weniger als 1
- 1 oder 2
- 3 oder 4
- 5 oder 6
- 7 oder 8
- 9 oder mehr

[1]Ein Standarddrink entspricht einem Glas Wein (ca. 1dl), einem Bier (ca. 3dl), einem Gläschen Spirituosen (ca. 4cl), einer Flasche Alkopops, einem Aperitif oder einem Longdrink. Ein grosses Bier (grosse Dose oder grosses Glas) entspricht 2 Standarddrinks, eine Flasche Wein 7 Standarddrinks.

Alkohol zur Verbesserung deiner Gehirnleistung

Diese Fragen betreffen deinen Konsum von **Alkohol** zur **Verbesserung deiner Gehirnleistung**. Also um bessere Leistungen zu erbringen, mit dem Schul- oder Arbeitsstress klarzukommen, um besser und länger lernen oder arbeiten zu können oder um vor der Prüfung oder einem Stresstag besser schlafen und entspannen zu können?

107 Wie häufig hast Du diese Substanz in den letzten 12 Monaten durchschnittlich verwendet, um deine Gehirnleistung für die Berufsvorbereitungsschule/Berufsfachschule/Kantonsschule zu verbessern?

Bitte wählen Sie nur eine der folgenden Antworten aus:

- Täglich
- Wöchentlich
- Alle 14 Tage
- Monatlich
- 1-2 Mal
- Nie

108 Wie häufig hast Du diese Substanz in den letzten 30 Tagen (ohne Urlaub) verwendet, um deine Gehirnleistung für die Berufsvorbereitungsschule/Berufsfachschule/Kantonsschule zu verbessern?

Bitte wählen Sie nur eine der folgenden Antworten aus:

- An 20 oder mehr Tagen
- An 10-19 Tagen
- An 4-9 Tagen
- An 1-3 Tagen
- Gar nicht

109 Was war der Grund für die Einnahme?

Bitte wählen Sie alle zutreffenden Antworten aus:

- [ ] Wach bleiben/länger lernen
- [ ] Sich konzentrieren/Aufmerksam sein
- [ ] Gedächtnis verbessern

- [ ] High sein (Rausch erzeugen)
- [ ] Gefühl geniessen
- [ ] Mit dem Druck klar kommen
- [ ] Besser schlafen können
- [ ] Allgemeine Entspannung
- [ ] Noten verbessern
- [ ] Prüfungsangst während der Prüfung bekämpfen
- [ ] Weil andere es tun
- [ ] Sonstiges:

110 Wurden Deine Erwartungen an die Wirkung der Substanz erfüllt?
Bitte wählen Sie nur eine der folgenden Antworten aus:

- ○ Ja
- ○ Nein
- ○ Teilweise
- ○ Ich weiss nicht

111 Würdest Du es in einer ähnlichen Situation wieder einnehmen?
Bitte wählen Sie nur eine der folgenden Antworten aus:

- ○ Ja
- ○ Nein
- ○ Vielleicht
- ○ Ich weiss nicht

Tabak in der Freizeit

Diese Fragen betreffen deinen Konsum von **Tabak** (zum Beispiel Zigaretten, Zigarren, Pfeife, Schnupftabak, Kautabak) in der **Freizeit**.

112 Wie häufig hast Du diese Substanz in den letzten 12 Monaten in der Freizeit z.B. im Ausgang, an Parties etc. durchschnittlich verwendet?
Bitte wählen Sie nur eine der folgenden Antworten aus:

- ○ Täglich
- ○ Wöchentlich
- ○ Alle 14 Tage
- ○ Monatlich
- ○ 1-2 Mal
- ○ Nie

113 Wie häufig hast Du diese Substanz in den letzten 30 Tagen in der Freizeit z.B. im Ausgang, an Parties etc. durchschnittlich verwendet?
Bitte wählen Sie nur eine der folgenden Antworten aus:

- ○ An 20 oder mehr Tagen
- ○ An 10-19 Tagen

○ An 4-9 Tagen
○ An 1-3 Tagen
○ Gar nicht

Tabak zur Verbesserung deiner Gehirnleistung

Diese Fragen betreffen deinen Konsum von **Tabak** (zum Beispiel Zigaretten, Zigarren, Pfeife, Schnupftabak, Kautabak) zur **Verbesserung deiner Gehirnleistung**. Also um bessere Leistungen zu erbringen, mit dem Schul- oder Arbeitsstress klarzukommen, um besser und länger lernen oder arbeiten zu können oder um vor der Prüfung oder einem Stresstag besser schlafen und entspannen zu können?

114 Wie häufig hast Du diese Substanz in den letzten 12 Monaten durchschnittlich verwendet, um deine Gehirnleistung für die Berufsvorbereitungsschule/Berufsfachschule/Kantonsschule zu verbessern?
Bitte wählen Sie nur eine der folgenden Antworten aus:

○ Täglich
○ Wöchentlich
○ Alle 14 Tage
○ Monatlich
○ 1-2 Mal
○ Nie

115 Wie häufig hast Du diese Substanz in den letzten 30 Tagen (ohne Urlaub) verwendet, um deine Gehirnleistung für die Berufsvorbereitungsschule/Berufsfachschule/Kantonsschule zu verbessern?
Bitte wählen Sie nur eine der folgenden Antworten aus:

○ An 20 oder mehr Tagen
○ An 10-19 Tagen
○ An 4-9 Tagen
○ An 1-3 Tagen
○ Gar nicht

116 Was war der Grund für die Einnahme?
Bitte wählen Sie alle zutreffenden Antworten aus:

☐ Wach bleiben/länger lernen
☐ Sich konzentrieren/Aufmerksam sein
☐ Gedächtnis verbessern
☐ High sein (Rausch erzeugen)
☐ Gefühl geniessen
☐ Mit dem Druck klar kommen
☐ Besser schlafen können
☐ Allgemeine Entspannung
☐ Noten verbessern
☐ Prüfungsangst während der Prüfung bekämpfen
☐ Weil andere es tun

☐ Sonstiges:

117 Wurden Deine Erwartungen an die Wirkung der Substanz erfüllt?
Bitte wählen Sie nur eine der folgenden Antworten aus:

- ○ Ja
- ○ Nein
- ○ Teilweise
- ○ Ich weiss nicht

118 Würdest Du es in einer ähnlichen Situation wieder einnehmen?
Bitte wählen Sie nur eine der folgenden Antworten aus:

- ○ Ja
- ○ Nein
- ○ Vielleicht
- ○ Ich weiss nicht

Hanfprodukte in der Freizeit

Diese Fragen betreffen deinen Konsum von **Hanfprodukte** (zum Beispiel Cannabis (Marihuana), Hasch, Haschöl) in der **Freizeit**.

119 Wie häufig hast Du diese Substanz in den letzten 12 Monaten in der Freizeit z.B. im Ausgang, an Parties etc. durchschnittlich verwendet?
Bitte wählen Sie nur eine der folgenden Antworten aus:

- ○ Täglich
- ○ Wöchentlich
- ○ Alle 14 Tage
- ○ Monatlich
- ○ 1-2 Mal
- ○ Nie

120 Wie häufig hast Du diese Substanz in den letzten 30 Tagen in der Freizeit z.B. im Ausgang, an Parties etc. durchschnittlich verwendet?
Bitte wählen Sie nur eine der folgenden Antworten aus:

- ○ An 20 oder mehr Tagen
- ○ An 10-19 Tagen
- ○ An 4-9 Tagen
- ○ An 1-3 Tagen
- ○ Gar nicht

Hanfprodukte zur Verbesserung deiner Gehirnleistung

Diese Fragen betreffen deinen Konsum von **Hanfprodukte** (zum Beispiel Cannabis (Marihuana), Hasch, Haschöl) zur **Verbesserung deiner Gehirnleistung**. Also um bessere Leistungen zu erbringen, mit dem Schul- oder Arbeitsstress klarzukommen, um besser und länger lernen oder arbeiten zu können oder um vor der Prüfung

oder einem Stresstag besser schlafen und entspannen zu können?

121 Wie häufig hast Du diese Substanz in den letzten 12 Monaten durchschnittlich verwendet, um deine Gehirnleistung für die Berufsvorbereitungsschule/Berufsfachschule/Kantonsschule zu verbessern?

Bitte wählen Sie nur eine der folgenden Antworten aus:

○ Täglich

○ Wöchentlich

○ Alle 14 Tage

○ Monatlich

○ 1-2 Mal

○ Nie

122 Wie häufig hast Du diese Substanz in den letzten 30 Tagen (ohne Urlaub) verwendet, um deine Gehirnleistung für die Berufsvorbereitungsschule/Berufsfachschule/Kantonsschule zu verbessern?

Bitte wählen Sie nur eine der folgenden Antworten aus:

○ An 20 oder mehr Tagen

○ An 10-19 Tagen

○ An 4-9 Tagen

○ An 1-3 Tagen

○ Gar nicht

123 Was war der Grund für die Einnahme?

Bitte wählen Sie alle zutreffenden Antworten aus:

☐ Wach bleiben/länger lernen

☐ Sich konzentrieren/Aufmerksam sein

☐ Gedächtnis verbessern

☐ High sein (Rausch erzeugen)

☐ Gefühl geniessen

☐ Mit dem Druck klar kommen

☐ Besser schlafen können

☐ Allgemeine Entspannung

☐ Noten verbessern

☐ Prüfungsangst während der Prüfung bekämpfen

☐ Weil andere es tun

☐ Sonstiges:

124 Wurden Deine Erwartungen an die Wirkung der Substanz erfüllt?

Bitte wählen Sie nur eine der folgenden Antworten aus:

○ Ja

○ Nein

○ Teilweise

○ Ich weiss nicht

125 Würdest Du es in einer ähnlichen Situation wieder einnehmen?
Bitte wählen Sie nur eine der folgenden Antworten aus:

- ○ Ja
- ○ Nein
- ○ Vielleicht
- ○ Ich weiss nicht

Kokain in der Freizeit

Diese Fragen betreffen deinen Konsum von **Kokain** in der **Freizeit**.

126 Wie häufig hast Du diese Substanz in den letzten 12 Monaten in der Freizeit z.B. im Ausgang, an Parties etc. durchschnittlich verwendet?
Bitte wählen Sie nur eine der folgenden Antworten aus:

- ○ Täglich
- ○ Wöchentlich
- ○ Alle 14 Tage
- ○ Monatlich
- ○ 1-2 Mal
- ○ Nie

127 Wie häufig hast Du diese Substanz in den letzten 30 Tagen in der Freizeit z.B. im Ausgang, an Parties etc. durchschnittlich verwendet?
Bitte wählen Sie nur eine der folgenden Antworten aus:

- ○ An 20 oder mehr Tagen
- ○ An 10-19 Tagen
- ○ An 4-9 Tagen
- ○ An 1-3 Tagen
- ○ Gar nicht

Kokain zur Verbesserung deiner Gehirnleistung

Diese Fragen betreffen deinen Konsum von **Kokain** zur **Verbesserung deiner Gehirnleistung**. Also um bessere Leistungen zu erbringen, mit dem Schul- oder Arbeitsstress klarzukommen, um besser und länger lernen oder arbeiten zu können oder um vor der Prüfung oder einem Stresstag besser schlafen und entspannen zu können?

128 Wie häufig hast Du diese Substanz in den letzten 12 Monaten durchschnittlich verwendet, um deine Gehirnleistung für die Berufsvorbereitungsschule/Berufsfachschule/Kantonsschule zu verbessern?
Bitte wählen Sie nur eine der folgenden Antworten aus:

- ○ Täglich
- ○ Wöchentlich
- ○ Alle 14 Tage
- ○ Monatlich
- ○ 1-2 Mal
- ○ Nie

129 Wie häufig hast Du diese Substanz in den letzten 30 Tagen (ohne Urlaub) verwendet, um deine Gehirnleistung für die Berufsvorbereitungsschule/Berufsfachschule/Kantonsschule zu verbessern?
Bitte wählen Sie nur eine der folgenden Antworten aus:

- ◯ An 20 oder mehr Tagen
- ◯ An 10-19 Tagen
- ◯ An 4-9 Tagen
- ◯ An 1-3 Tagen
- ◯ Gar nicht

130 Was war der Grund für die Einnahme?
Bitte wählen Sie alle zutreffenden Antworten aus:

- ☐ Wach bleiben/länger lernen
- ☐ Sich konzentrieren/Aufmerksam sein
- ☐ Gedächtnis verbessern
- ☐ High sein (Rausch erzeugen)
- ☐ Gefühl geniessen
- ☐ Mit dem Druck klar kommen
- ☐ Besser schlafen können
- ☐ Allgemeine Entspannung
- ☐ Noten verbessern
- ☐ Prüfungsangst während der Prüfung bekämpfen
- ☐ Weil andere es tun
- ☐ Sonstiges:

131 Wurden Deine Erwartungen an die Wirkung der Substanz erfüllt?
Bitte wählen Sie nur eine der folgenden Antworten aus:

- ◯ Ja
- ◯ Nein
- ◯ Teilweise
- ◯ Ich weiss nicht

132 Würdest Du es in einer ähnlichen Situation wieder einnehmen?
Bitte wählen Sie nur eine der folgenden Antworten aus:

- ◯ Ja
- ◯ Nein
- ◯ Vielleicht
- ◯ Ich weiss nicht

Amphetamine in der Freizeit

Diese Fragen betreffen deinen Konsum von **Amphetamine** (Speed) in der **Freizeit**.

133 Wie häufig hast Du diese Substanz in den letzten 12 Monaten in der Freizeit z.B. im Ausgang, an

Parties etc. durchschnittlich verwendet?
Bitte wählen Sie nur eine der folgenden Antworten aus:

- Täglich
- Wöchentlich
- Alle 14 Tage
- Monatlich
- 1-2 Mal
- Nie

134 Wie häufig hast Du diese Substanz in den letzten 30 Tagen in der Freizeit z.B. im Ausgang, an Parties etc. durchschnittlich verwendet?
Bitte wählen Sie nur eine der folgenden Antworten aus:

- An 20 oder mehr Tagen
- An 10-19 Tagen
- An 4-9 Tagen
- An 1-3 Tagen
- Gar nicht

Amphetamine zur Verbesserung deiner Gehirnleistung

Diese Fragen betreffen deinen Konsum von **Amphetamine** (Speed) zur **Verbesserung deiner Gehirnleistung**. Also um bessere Leistungen zu erbringen, mit dem Schul- oder Arbeitsstress klarzukommen, um besser und länger lernen oder arbeiten zu können oder um vor der Prüfung oder einem Stresstag besser schlafen und entspannen zu können?

135 Wie häufig hast Du diese Substanz in den letzten 12 Monaten durchschnittlich verwendet, um deine Gehirnleistung für die Berufsvorbereitungsschule/Berufsfachschule/Kantonsschule zu verbessern?
Bitte wählen Sie nur eine der folgenden Antworten aus:

- Täglich
- Wöchentlich
- Alle 14 Tage
- Monatlich
- 1-2 Mal
- Nie

136 Wie häufig hast Du diese Substanz in den letzten 30 Tagen (ohne Urlaub) verwendet, um deine Gehirnleistung für die Berufsvorbereitungsschule/Berufsfachschule/Kantonsschule zu verbessern?
Bitte wählen Sie nur eine der folgenden Antworten aus:

- An 20 oder mehr Tagen
- An 10-19 Tagen
- An 4-9 Tagen
- An 1-3 Tagen
- Gar nicht

137 Was war der Grund für die Einnahme?

Bitte wählen Sie alle zutreffenden Antworten aus:

- ☐ Wach bleiben/länger lernen
- ☐ Sich konzentrieren/Aufmerksam sein
- ☐ Gedächtnis verbessern
- ☐ High sein (Rausch erzeugen)
- ☐ Gefühl geniessen
- ☐ Mit dem Druck klar kommen
- ☐ Besser schlafen können
- ☐ Allgemeine Entspannung
- ☐ Noten verbessern
- ☐ Prüfungsangst während der Prüfung bekämpfen
- ☐ Weil andere es tun
- ☐ Sonstiges:

138 Wurden Deine Erwartungen an die Wirkung der Substanz erfüllt?
Bitte wählen Sie nur eine der folgenden Antworten aus:

- ○ Ja
- ○ Nein
- ○ Teilweise
- ○ Ich weiss nicht

139 Würdest Du es in einer ähnlichen Situation wieder einnehmen?
Bitte wählen Sie nur eine der folgenden Antworten aus:

- ○ Ja
- ○ Nein
- ○ Vielleicht
- ○ Ich weiss nicht

Ecstasy (MDMA) in der Freizeit

Diese Fragen betreffen deinen Konsum von **Ecstasy** (MDMA) in der **Freizeit**.
140 Wie häufig hast Du diese Substanz in den letzten 12 Monaten in der Freizeit z.B. im Ausgang, an Parties etc. durchschnittlich verwendet?
Bitte wählen Sie nur eine der folgenden Antworten aus:

- ○ Täglich
- ○ Wöchentlich
- ○ Alle 14 Tage
- ○ Monatlich
- ○ 1-2 Mal
- ○ Nie

141 Wie häufig hast Du diese Substanz in den letzten 30 Tagen in der Freizeit z.B. im Ausgang, an Parties etc. durchschnittlich verwendet?

Bitte wählen Sie nur eine der folgenden Antworten aus:

- ○ An 20 oder mehr Tagen
- ○ An 10-19 Tagen
- ○ An 4-9 Tagen
- ○ An 1-3 Tagen
- ○ Gar nicht

Ecstasy (MDMA) zur Verbesserung deiner Gehirnleistung

Diese Fragen betreffen deinen Konsum von **Ecstasy** (MDMA) zur **Verbesserung deiner Gehirnleistung**. Also um bessere Leistungen zu erbringen, mit dem Schul- oder Arbeitsstress klarzukommen, um besser und länger lernen oder arbeiten zu können oder um vor der Prüfung oder einem Stresstag besser schlafen und entspannen zu können?

142 Wie häufig hast Du diese Substanz in den letzten 12 Monaten durchschnittlich verwendet, um deine Gehirnleistung für die Berufsvorbereitungsschule/Berufsfachschule/Kantonsschule zu verbessern?
Bitte wählen Sie nur eine der folgenden Antworten aus:

- ○ Täglich
- ○ Wöchentlich
- ○ Alle 14 Tage
- ○ Monatlich
- ○ 1-2 Mal
- ○ Nie

143 Wie häufig hast Du diese Substanz in den letzten 30 Tagen (ohne Urlaub) verwendet, um deine Gehirnleistung für die Berufsvorbereitungsschule/Berufsfachschule/Kantonsschule zu verbessern?
Bitte wählen Sie nur eine der folgenden Antworten aus:

- ○ An 20 oder mehr Tagen
- ○ An 10-19 Tagen
- ○ An 4-9 Tagen
- ○ An 1-3 Tagen
- ○ Gar nicht

144 Was war der Grund für die Einnahme?
Bitte wählen Sie alle zutreffenden Antworten aus:

- ☐ Wach bleiben/länger lernen
- ☐ Sich konzentrieren/Aufmerksam sein
- ☐ Gedächtnis verbessern
- ☐ High sein (Rausch erzeugen)
- ☐ Gefühl geniessen
- ☐ Mit dem Druck klar kommen
- ☐ Besser schlafen können
- ☐ Allgemeine Entspannung
- ☐ Noten verbessern

☐ Prüfungsangst während der Prüfung bekämpfen

☐ Weil andere es tun

☐ Sonstiges:

145 Wurden Deine Erwartungen an die Wirkung der Substanz erfüllt?
Bitte wählen Sie nur eine der folgenden Antworten aus:

○ Ja

○ Nein

○ Teilweise

○ Ich weiss nicht

146 Würdest Du es in einer ähnlichen Situation wieder einnehmen?
Bitte wählen Sie nur eine der folgenden Antworten aus:

○ Ja

○ Nein

○ Vielleicht

○ Ich weiss nicht

Kaffee in der Freizeit

Diese Fragen betreffen deinen Konsum von **Kaffee** in der **Freizeit**.

147 Wie häufig hast Du diese Substanz in den letzten 12 Monaten in der Freizeit z.B. im Ausgang, an Parties etc. durchschnittlich verwendet?
Bitte wählen Sie nur eine der folgenden Antworten aus:

○ Täglich

○ Wöchentlich

○ Alle 14 Tage

○ Monatlich

○ 1-2 Mal

○ Nie

148 Wie häufig hast Du diese Substanz in den letzten 30 Tagen in der Freizeit z.B. im Ausgang, an Parties etc. durchschnittlich verwendet?
Bitte wählen Sie nur eine der folgenden Antworten aus:

○ An 20 oder mehr Tagen

○ An 10-19 Tagen

○ An 4-9 Tagen

○ An 1-3 Tagen

○ Gar nicht

Kaffee zur Verbesserung deiner Gehirnleistung

Diese Fragen betreffen deinen Konsum von **Kaffee** zur **Verbesserung deiner Gehirnleistung**. Also um bessere

Leistungen zu erbringen, mit dem Schul- oder Arbeitsstress klarzukommen, um besser und länger lernen oder arbeiten zu können oder um vor der Prüfung oder einem Stresstag besser schlafen und entspannen zu können?

149 Wie häufig hast Du diese Substanz in den letzten 12 Monaten durchschnittlich verwendet, um deine Gehirnleistung für die Berufsvorbereitungsschule/Berufsfachschule/Kantonsschule zu verbessern?

Bitte wählen Sie nur eine der folgenden Antworten aus:

- ○ Täglich
- ○ Wöchentlich
- ○ Alle 14 Tage
- ○ Monatlich
- ○ 1-2 Mal
- ○ Nie

150 Wie häufig hast Du diese Substanz in den letzten 30 Tagen (ohne Urlaub) verwendet, um deine Gehirnleistung für die Berufsvorbereitungsschule/Berufsfachschule/Kantonsschule zu verbessern?

Bitte wählen Sie nur eine der folgenden Antworten aus:

- ○ An 20 oder mehr Tagen
- ○ An 10-19 Tagen
- ○ An 4-9 Tagen
- ○ An 1-3 Tagen
- ○ Gar nicht

151 Was war der Grund für die Einnahme?

Bitte wählen Sie alle zutreffenden Antworten aus:

- ☐ Wach bleiben/länger lernen
- ☐ Sich konzentrieren/Aufmerksam sein
- ☐ Gedächtnis verbessern
- ☐ High sein (Rausch erzeugen)
- ☐ Gefühl geniessen
- ☐ Mit dem Druck klar kommen
- ☐ Besser schlafen können
- ☐ Allgemeine Entspannung
- ☐ Noten verbessern
- ☐ Prüfungsangst während der Prüfung bekämpfen
- ☐ Weil andere es tun
- ☐ Sonstiges:

152 Wurden Deine Erwartungen an die Wirkung der Substanz erfüllt?

Bitte wählen Sie nur eine der folgenden Antworten aus:

- ○ Ja
- ○ Nein
- ○ Teilweise
- ○ Ich weiss nicht

153 Würdest Du es in einer ähnlichen Situation wieder einnehmen?
Bitte wählen Sie nur eine der folgenden Antworten aus:

- Ja
- Nein
- Vielleicht
- Ich weiss nicht

Koffeintabletten in der Freizeit

Diese Fragen betreffen deinen Konsum von **Koffeintabletten** (zum Beispiel Coffeinum N®, Percoffedrinol®) in der **Freizeit**.

154 Wie häufig hast Du diese Substanz in den letzten 12 Monaten in der Freizeit z.B. im Ausgang, an Parties etc. durchschnittlich verwendet?
Bitte wählen Sie nur eine der folgenden Antworten aus:

- Täglich
- Wöchentlich
- Alle 14 Tage
- Monatlich
- 1-2 Mal
- Nie

155 Wie häufig hast Du diese Substanz in den letzten 30 Tagen in der Freizeit z.B. im Ausgang, an Parties etc. durchschnittlich verwendet?
Bitte wählen Sie nur eine der folgenden Antworten aus:

- An 20 oder mehr Tagen
- An 10-19 Tagen
- An 4-9 Tagen
- An 1-3 Tagen
- Gar nicht

Koffeintabletten zur Verbesserung deiner Gehirnleistung

Diese Fragen betreffen deinen Konsum von **Koffeintabletten** (zum Beispiel Coffeinum N®, Percoffedrinol®) zur **Verbesserung deiner Gehirnleistung**. Also um bessere Leistungen zu erbringen, mit dem Schul- oder Arbeitsstress klarzukommen, um besser und länger lernen oder arbeiten zu können oder um vor der Prüfung oder einem Stresstag besser schlafen und entspannen zu können?

156 Wie häufig hast Du diese Substanz in den letzten 12 Monaten durchschnittlich verwendet, um deine Gehirnleistung für die Berufsvorbereitungsschule/Berufsfachschule/Kantonsschule zu verbessern?
Bitte wählen Sie nur eine der folgenden Antworten aus:

- Täglich
- Wöchentlich
- Alle 14 Tage
- Monatlich

○ 1-2 Mal
○ Nie

157 Wie häufig hast Du diese Substanz in den letzten 30 Tagen (ohne Urlaub) verwendet, um deine Gehirnleistung für die Berufsvorbereitungsschule/Berufsfachschule/Kantonsschule zu verbessern?
Bitte wählen Sie nur eine der folgenden Antworten aus:
○ An 20 oder mehr Tagen
○ An 10-19 Tagen
○ An 4-9 Tagen
○ An 1-3 Tagen
○ Gar nicht

158 Was war der Grund für die Einnahme?
Bitte wählen Sie alle zutreffenden Antworten aus:
☐ Wach bleiben/länger lernen
☐ Sich konzentrieren/Aufmerksam sein
☐ Gedächtnis verbessern
☐ High sein (Rausch erzeugen)
☐ Gefühl geniessen
☐ Mit dem Druck klar kommen
☐ Besser schlafen können
☐ Allgemeine Entspannung
☐ Noten verbessern
☐ Prüfungsangst während der Prüfung bekämpfen
☐ Weil andere es tun
☐ Sonstiges:

159 Wurden Deine Erwartungen an die Wirkung der Substanz erfüllt?
Bitte wählen Sie nur eine der folgenden Antworten aus:
○ Ja
○ Nein
○ Teilweise
○ Ich weiss nicht

160 Würdest Du es in einer ähnlichen Situation wieder einnehmen?
Bitte wählen Sie nur eine der folgenden Antworten aus:
○ Ja
○ Nein
○ Vielleicht
○ Ich weiss nicht

Energy Drinks in der Freizeit

Diese Fragen betreffen deinen Konsum von **Energy Drinks** (zum Beispiel Red Bull®, guaranahaltige Getränke) in der **Freizeit**.

161 Wie häufig hast Du diese Substanz in den letzten 12 Monaten in der Freizeit z.B. im Ausgang, an Parties etc. durchschnittlich verwendet?
Bitte wählen Sie nur eine der folgenden Antworten aus:

- ◯ Täglich
- ◯ Wöchentlich
- ◯ Alle 14 Tage
- ◯ Monatlich
- ◯ 1-2 Mal
- ◯ Nie

162 Wie häufig hast Du diese Substanz in den letzten 30 Tagen in der Freizeit z.B. im Ausgang, an Parties etc. durchschnittlich verwendet?
Bitte wählen Sie nur eine der folgenden Antworten aus:

- ◯ An 20 oder mehr Tagen
- ◯ An 10-19 Tagen
- ◯ An 4-9 Tagen
- ◯ An 1-3 Tagen
- ◯ Gar nicht

Energy Drinks zur Verbesserung deiner Gehirnleistung

Diese Fragen betreffen deinen Konsum von **Energy Drinks** (zum Beispiel Red Bull®, guaranahaltige Getränke) zur **Verbesserung deiner Gehirnleistung**. Also um bessere Leistungen zu erbringen, mit dem Schul- oder Arbeitsstress klarzukommen, um besser und länger lernen oder arbeiten zu können oder um vor der Prüfung oder einem Stresstag besser schlafen und entspannen zu können?

163 Wie häufig hast Du diese Substanz in den letzten 12 Monaten durchschnittlich verwendet, um deine Gehirnleistung für die Berufsvorbereitungsschule/Berufsfachschule/Kantonsschule zu verbessern?
Bitte wählen Sie nur eine der folgenden Antworten aus:

- ◯ Täglich
- ◯ Wöchentlich
- ◯ Alle 14 Tage
- ◯ Monatlich
- ◯ 1-2 Mal
- ◯ Nie

164 Wie häufig hast Du diese Substanz in den letzten 30 Tagen (ohne Urlaub) verwendet, um deine Gehirnleistung für die Berufsvorbereitungsschule/Berufsfachschule/Kantonsschule zu verbessern?
Bitte wählen Sie nur eine der folgenden Antworten aus:

- ◯ An 20 oder mehr Tagen
- ◯ An 10-19 Tagen
- ◯ An 4-9 Tagen

○ An 1-3 Tagen
○ Gar nicht

165 Was war der Grund für die Einnahme?
Bitte wählen Sie alle zutreffenden Antworten aus:

☐ Wach bleiben/länger lernen
☐ Sich konzentrieren/Aufmerksam sein
☐ Gedächtnis verbessern
☐ High sein (Rausch erzeugen)
☐ Gefühl geniessen
☐ Mit dem Druck klar kommen
☐ Besser schlafen können
☐ Allgemeine Entspannung
☐ Noten verbessern
☐ Prüfungsangst während der Prüfung bekämpfen
☐ Weil andere es tun
☐ Sonstiges:

166 Wurden Deine Erwartungen an die Wirkung der Substanz erfüllt?
Bitte wählen Sie nur eine der folgenden Antworten aus:

○ Ja
○ Nein
○ Teilweise
○ Ich weiss nicht

167 Würdest Du es in einer ähnlichen Situation wieder einnehmen?
Bitte wählen Sie nur eine der folgenden Antworten aus:

○ Ja
○ Nein
○ Vielleicht
○ Ich weiss nicht

Andere Substanz in der Freizeit

Diese Fragen betreffen deinen Konsum einer **anderen Substanz** in der **Freizeit**.

168 Um welche Substanz(en) handelt es sich?
Bitte geben Sie Ihre Antwort hier ein:

169 Wie häufig hast Du diese Substanz in den letzten 12 Monaten in der Freizeit z.B. im Ausgang, an Parties etc. durchschnittlich verwendet?
Bitte wählen Sie nur eine der folgenden Antworten aus:

○ Täglich
○ Wöchentlich

- Alle 14 Tage
- Monatlich
- 1-2 Mal
- Nie

170 Wie häufig hast Du diese Substanz in den letzten 30 Tagen in der Freizeit z.B. im Ausgang, an Parties etc. durchschnittlich verwendet?
Bitte wählen Sie nur eine der folgenden Antworten aus:

- An 20 oder mehr Tagen
- An 10-19 Tagen
- An 4-9 Tagen
- An 1-3 Tagen
- Gar nicht

Andere Substanz zur Verbesserung deiner Gehirnleistung

Diese Fragen betreffen deinen Konsum einer **anderen Substanz** zur **Verbesserung deiner Gehirnleistung**. Also um bessere Leistungen zu erbringen, mit dem Schul- oder Arbeitsstress klarzukommen, um besser und länger lernen oder arbeiten zu können oder um vor der Prüfung oder einem Stresstag besser schlafen und entspannen zu können?

171 Um welche Substanz(en) handelt es sich?
Bitte geben Sie Ihre Antwort hier ein:

172 Wie häufig hast Du diese Substanz in den letzten 12 Monaten durchschnittlich verwendet, um deine Gehirnleistung für die Berufsvorbereitungsschule/Berufsfachschule/Kantonsschule zu verbessern?
Bitte wählen Sie nur eine der folgenden Antworten aus:

- Täglich
- Wöchentlich
- Alle 14 Tage
- Monatlich
- 1-2 Mal
- Nie

173 Wie häufig hast Du diese Substanz in den letzten 30 Tagen (ohne Urlaub) verwendet, um deine Gehirnleistung für die Berufsvorbereitungsschule/Berufsfachschule/Kantonsschule zu verbessern?
Bitte wählen Sie nur eine der folgenden Antworten aus:

- An 20 oder mehr Tagen
- An 10-19 Tagen
- An 4-9 Tagen
- An 1-3 Tagen
- Gar nicht

174 Was war der Grund für die Einnahme?
Bitte wählen Sie alle zutreffenden Antworten aus:

☐ Wach bleiben/länger lernen
☐ Sich konzentrieren/Aufmerksam sein
☐ Gedächtnis verbessern
☐ High sein (Rausch erzeugen)
☐ Gefühl geniessen
☐ Mit dem Druck klar kommen
☐ Besser schlafen können
☐ Allgemeine Entspannung
☐ Noten verbessern
☐ Prüfungsangst während der Prüfung bekämpfen
☐ Weil andere es tun
☐ Sonstiges:

175 Wurden Deine Erwartungen an die Wirkung der Substanz erfüllt?
Bitte wählen Sie nur eine der folgenden Antworten aus:

○ Ja
○ Nein
○ Teilweise
○ Ich weiss nicht

176 Würdest Du es in einer ähnlichen Situation wieder einnehmen?
Bitte wählen Sie nur eine der folgenden Antworten aus:

○ Ja
○ Nein
○ Vielleicht
○ Ich weiss nicht

Nutzung und Bezug

177 Kennst Du Personen aus deinem Familien- und/oder Bekanntenkreis, die rezeptpflichtige Medikamente ohne medizinischen Grund und/oder psychoaktive Substanzen (wie zum Beispiel Cannabis, Kokain, Ecstasy etc.) konsumieren, um ihre Gehirnleistung zu verbessern?
Bitte wählen Sie nur eine der folgenden Antworten aus:

○ Ja
○ Nein

178 Von welcher Quelle beziehst Du rezeptpflichtige Medikamente, die Du zur Verbesserung deiner Gehirnleistung für die Schule einsetzen willst/wolltest?
Bitte wählen Sie nur eine der folgenden Antworten aus:

○ Internet
○ Arzt/Ärztin
○ Pädiater/Pädiaterin bzw. Kinderarzt/Kinderärztin
○ Psychiater/Psychiaterin

- ○ Apotheker/Apothekerin
- ○ Von jemandem, der das Medikament vom Arzt verschrieben bekam
- ○ Dealer
- ○ Eltern
- ○ Kollege/Kollegin
- ○ Andere Personen
- ○ Ich beziehe keine verschreibungspflichtigen Medikamente zur Verbesserung der Gehirnleistung

179 In welchen Situationen hast du die Substanz(en) zum Hirndoping hauptsächlich eingenommen oder nimmst Du die Substanz(en) hauptsächlich ein?
Bitte wählen Sie nur eine der folgenden Antworten aus:

- ○ Prüfungsvorbereitung
- ○ Prüfungssituation
- ○ Allgemein bei Stress
- ○ Sonstiges

Moralische Fragen

180 Bist du grundsätzlich einverstanden damit, dass Schüler/innen Medikamente und/oder psychoaktive Substanzen (wie zum Beispiel Cannabis, Kokain, Ecstasy etc.) konsumieren um Ihre Gehirnleistung zu verbessern?
Bitte wählen Sie nur eine der folgenden Antworten aus:

- ○ Ja
- ○ Nein

181 Gibt es für Dich persönlich gute Gründe, die den Konsum von Medikamenten und/oder psychoaktiven Substanzen (wie zum Beispiel Cannabis, Kokain, Ecstasy etc.) zur Verbesserung der Gehirnleistung für die Schule entschuldigen können? (mehrere Antworten möglich)
Bitte wählen Sie alle zutreffenden Antworten aus:

- ☐ Aufmerksamkeit / Konzentration / Gedächtnisleistung steigern
- ☐ Müdigkeit unterdrücken
- ☐ Schlafstörungen
- ☐ Termindruck
- ☐ Konflikte
- ☐ Konkurrenzdruck
- ☐ Nervosität / Lampenfieber
- ☐ Schlechte Stimmung
- ☐ Schmerzen
- ☐ Freundlichere Ausstrahlung
- ☐ Depression / psychische Erkrankung
- ☐ Grippe / Erkältung / Fieber
- ☐ Ärztliche Empfehlung
- ☐ Nein, es gibt keine vertretbaren Gründe

☐ Sonstiges:

Gesundheitszustand

182 Warst Du jemals wegen einer oder mehrerer der folgenden psychischen Erkrankungen in therapeutischer/ärztlicher Behandlung bzw. wurde bei Dir bereits jemals eine der folgenden psychischen Erkrankungen diagnostiziert/festgestellt oder?
Bitte wählen Sie alle zutreffenden Antworten aus:

☐ Keine

☐ ADS oder ADHS

☐ Depressive Störung/Depression

☐ Zwangsstörung

☐ Angststörung

☐ Persönlichkeitsstörung

☐ Posttraumatische Belastungsstörung

☐ Suchterkrankung

☐ andere psychische Erkrankung, nämlich:

183 Wurde bei Dir bereits eine oder mehrere der folgenden körperlichen Erkrankungen diagnostiziert/festgestellt oder warst Du deswegen in ärztlicher Behandlung
Bitte wählen Sie alle zutreffenden Antworten aus:

☐ Keine

☐ Herzprobleme

☐ Lungen-/Atembescherden

☐ Schlaferkrankung

☐ Krebs

☐ Andere Langzeiterkrankung, nämlich:

184 Nimmst Du oder hast Du früher aufgrund der Erkrankung regelmässig Medikamente eingenommen?
Bitte wählen Sie nur eine der folgenden Antworten aus:

○ Ja

○ Nein

185 Welche Medikamente hast Du eingenommen?
Beantworten Sie diese Frage nur, wenn folgende Bedingungen erfüllt sind:
Antwort war 'Ja' bei Frage 184 (Nimmst Du oder hast Du früher aufgrund der Erkrankung regelmässig Medikamente eingenommen?)
Bitte geben Sie Ihre Antwort hier ein:

186 Hast Du das Medikament schon mal anders (d.h. zeitlich verschoben, in einer anderen Menge oder über einen anderen Einnahmeweg wie z. B. geschnupft anstatt geschluckt) als ärztlich verschrieben eingenommen, um deine schulischen Leistungen zu verbessern?

Beantworten Sie diese Frage nur, wenn folgende Bedingungen erfüllt sind:
Antwort war 'Ja' bei Frage 184 (Nimmst Du oder hast Du früher aufgrund der Erkrankung regelmässig Medikamente eingenommen?)

Bitte wählen Sie nur eine der folgenden Antworten aus:

- ◯ Ja
- ◯ Nein

Liebe Schülerin, lieber Schüler

Gerne möchten wie Dir noch wichtige Informationen zur Studie mitgeben.
Wie bereits anfangs erläutert, zeigt die Forschung, dass gewisse Schüler/innen und Studierende zu Medikamenten und/oder anderen Substanzen greifen, um Hirndoping zu betreiben.
Dies wird getan, obwohl die **Wirksamkeit** der Hirndoping-Substanzen bislang **nicht erwiesen** ist.
Viele Studien, die bis anhin durchgeführt wurden, zeigen, dass der Konsum der Hirndoping-Substanzen **keine** Verbesserung der geistigen Leistungsfähigkeit bei gesunden Menschen herbeiführt. Manche Studien konnten sogar zeigen, dass der Konsum von Hirndoping-Substanzen mit einer **Verschlechterung** der geistigen Leistungsfähigkeit verbunden ist.
Hinzu kommt, dass die Einnahme vieler Substanzen mit **Nebenwirkungen** verbunden ist. Solche Nebeneffekte können schwerwiegend sein. Sie können je nach Substanz zum Beispiel als Kopfschmerzen, Übelkeit, Schlafstörungen, Bluthochdruck, Wachstumshemmung, Angstzustände, Herzrasen, neurologische Störungen, Hypersensibilität oder Suchtprobleme auftreten.

Das Fazit lautet deshalb: Hirndoping bringt wenig oder nichts und ist riskant!

Wir danken Dir herzlich für deine Mitarbeit!

Bei Fragen oder Anmerkungen zur Studie stehen wir Dir gerne zur Verfügung: hirndopingfragen@hotmail.com

Projektleitung:
PD Dr. Michael Schaub, Direktor Schweizer Institut für Sucht- und Gesundheitsforschung; Domenic Schnoz, lic. phil., Leiter Zürcher Fachstelle für Alkohol- und Medikamentenprobleme; Vigeli Venzin, Leiter Fachstelle Suchtprävention Mittelschulen und Berufsbildung

Vielen Dank für die Beantwortung des Fragebogens!
